# Synthesis biological evaluation and molecular docking of isatin hybrids as anti-cancer and anti-microbial agents

**DOI:** 10.1080/14756366.2023.2288548

**Published:** 2023-12-11

**Authors:** Mohammad Altamimi, Saeed Ali Syed, Burak Tuzun, Mohammad Rashid Alhazani, Osamah Alnemer, Ahmed Bari

**Affiliations:** a Department of Pharmaceutics, College of Pharmacy, King Saud University, Riyadh, Saudi Arabia; b Department of Pharmaceutical Chemistry, College of Pharmacy, King Saud University, Riyadh, Saudi Arabia; c Plant and Animal Production Department, Technical Sciences Vocational School of Sivas, Sivas Cumhuriyet University, Sivas, Turkey; d College of Pharmacy, Central Laboratory, King Saud University, Riyadh, Saudi Arabia

**Keywords:** Hydrazone–indolinone, antimicrobial, anticancer, oxindole, spiroxindole

## Abstract

Isatin, known as 1*H*-indole-2,3-dione, was originally recognised as a synthetic molecule until its discovery in the fruits of the cannonball tree, *Couroupita guianensis*. It is naturally occurring in plants of the genus *Isatis* and serves as a metabolic derivative of adrenaline in humans. Isatin possesses significant pharmacological importance, and its synthetic versatility has prompted extensive interest in its derivative compounds due to their diverse biological and pharmacological properties. These derivatives represent a valuable class of heterocyclic compounds with potential applications as precursors for synthesizing numerous valuable drugs. In the pursuit of advancing our research on isatin hybrids, we investigate the utilisation of readily available hydrazonoindolin-2-one and isatin as starting materials for the synthesis of a wide range of analogues. Characterisation of the synthesized compounds was carried out through various analytical techniques. Furthermore, the obtained compounds were subjected to extensive testing to evaluate their anticancer and antimicrobial activities. Specifically, their efficacy against key proteins, namely *Staphylococcus aureus* protein (PDB ID: 1JIJ), *Escherichia coli* protein (PDB ID: 1T9U), *Pseudomonas aeruginosa* protein (PDB ID: 2UV0), and *Acinetobacter baumannii* protein (PDB ID: 4HKG), was examined through molecular docking calculations. Several molecules, such as 3, 4, 6, 16, and 19, displayed remarkable activity against the renal cancer cell line UO-31. Additionally, the results of antimicrobial activity testing revealed that compound **16** exhibited significant cytotoxicity against *Candida albicans* and *Cryptococcus neoformans*. Subsequently, ADME/T calculations were performed to gain insights into the potential effects and reactions of these molecules within human metabolism. This comprehensive study provides valuable insights into the potential pharmacological applications of isatin derivatives and underscores their significance in drug development.

## Introduction

Isatin and its analogues exhibit remarkable versatility as synthetic compounds, serving as valuable precursors for the production of numerous pharmaceuticals currently available in the market^1^. Isatin, an indole derivative, occurs naturally in plants and constitutes a component of the secretion produced by the parotid gland of bufo frogs. Natural occurrences of substituted isatin compounds also exist; for instance, methoxyphenyl pentylisatin has been extracted from the tumorigenic plant *Melochia tomentosa* in the Caribbean. Furthermore, various substituted isatins have been discovered in fungi, with methylbuten-2-yl-isatin being isolated from *Streptomyces albus* and *Chaetomium globosum*^2–4^. Derivatives of isatin constitute an essential faction of heterocyclic chemistry, and their biological potential has been an exciting field for their pharmacological properties. These activities include analgesic, anticancer, anti-inflammatory, antitubercular, antimicrobial, antifungal, and antiviral^5–7^.

**Figure 1.**
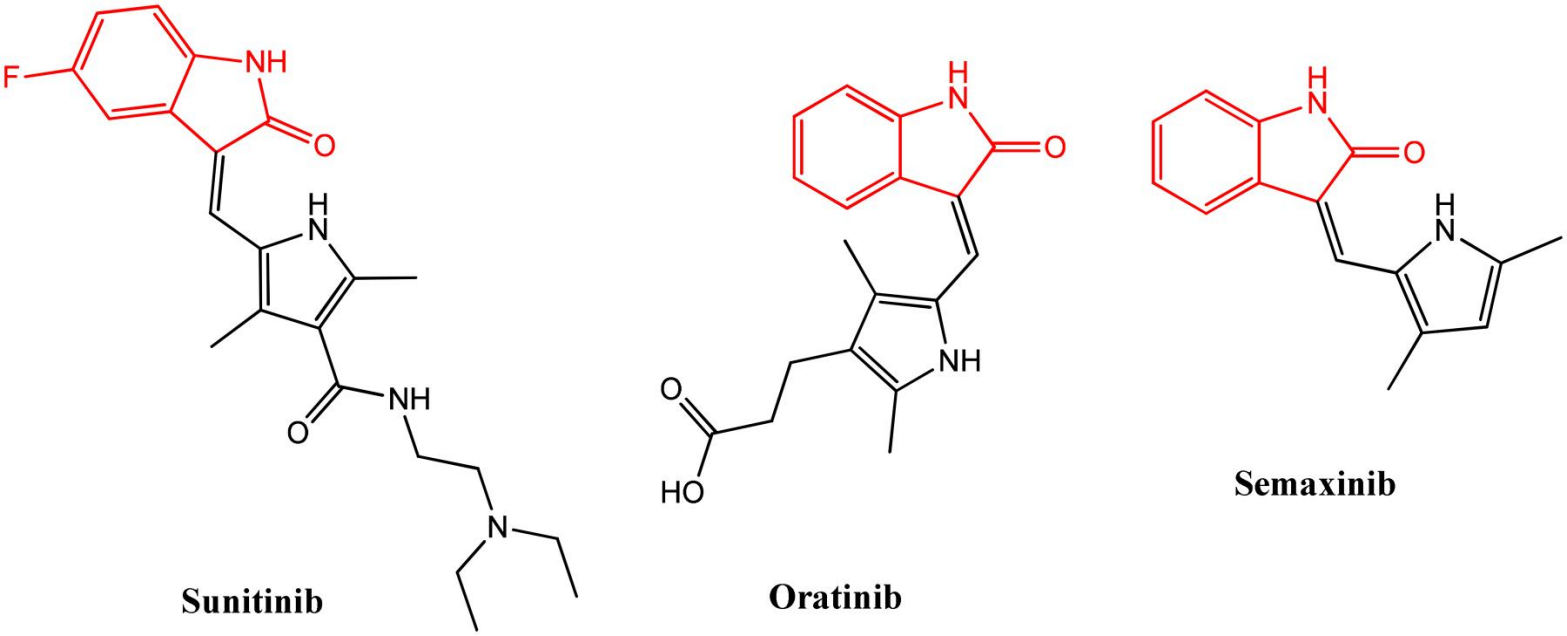
Structures of isatin based compounds.

The isatin skeleton attached to heterocyclic rings is a privileged core in numerous molecules available in the market (Figure 1). Interestingly, oxazolidine, thiazolidine, and other fused ring systems attached to isatin exhibited promising antitumor activities with different modes of action^8–10^. Despite the extensive progress on isatin and its derivatives, the development methods with pharmacological profiles and therapeutic safety represent a significant concern for many scientists. Due to the wide range of properties, heterocyclic chemistry has generated intensive interest over the last few years. Several reports on synthesizing heterocyclic molecules with anticancer and anti-microbial potential have been published so far^11^^,^^12^. Drug resistance along with clinical experience confirms that single targets might not always provide the preferred biological outcome in cancer chemotherapy. This is due to the development of resistance by self-modification of the target through mutation. Moreover, among the large number of compounds synthesized and screened for their potential *in vitro* antiproliferative agents, derivatives possessing the benzothiophene, 9*H* fluorine, and dihydrobenzodioxane structural description hybridized with isatin moiety have gained significant attention for current medicinal chemists^13^. In recent periods, the molecular hybrid approach has resulted in many new and interesting chemical entities with better antitumor activity, selectivity, and bridged side effects. A recent report suggested the use of 4-oxadiazoles and its derivatives as potent anticancer agents against *MCF-7* cells^14–16^. Moreover, in another report by Gong and co-workers, the design, synthesis, and SAR of diarylthiosemicarbazide derivatives as potent antiproliferative agents against various cancer cell lines were published^17^. Due to the major limitations such as multi-drug resistance and drug-related toxicity, several cutting-edge chemotherapies are unsuccessful. Therefore, the demand for newer and safe anticancer drugs with high efficiency and low toxicity for future cancer chemotherapy is highly desirable.

Theoretical calculations represent a vital computational approach employed to predict molecule activities and expedite the development and enhancement of active sites. Such calculations significantly shorten the process of designing more effective and potent molecules. In this context, diverse levels and base sets of ligand and metal complexes were employed in theoretical calculations to assess the activities of the molecules. Despite substantial progress in the synthesis of 1*H*-indole-2,3-dione analogues and our ongoing interest in developing an efficient methodology for synthesizing fine chemicals and pharmaceutically relevant heterocyclic compounds, this study focuses on synthesizing isatin scaffolds coupled with various privileged heterocyclic molecules to identify potent nuclei. Within the scope of this paper, we also investigate the interaction of these molecules with *Aspergillus niger* protein, identifying the most adherent molecules. Additionally, the activities of each studied molecule on the HF/6-31g basis set are probed using Gaussian calculations. These calculations provide valuable insights into their activities through various parameters. Furthermore, molecular docking calculations are employed to evaluate the activities of these molecules against specific proteins, including *Staphylococcus aureus* protein (PDB ID: 1JIJ), *Escherichia coli* protein (PDB ID: 1T9U), *Pseudomonas aeruginosa* protein (PDB ID: 2UV0), and *Acinetobacter baumannii* protein (PDB ID: 4HKG). Subsequently, ADME/T calculations are performed to assess the effects and reactions of these molecules within the context of human metabolism.

## Results and discussion

### Chemistry

**Figure 2.**
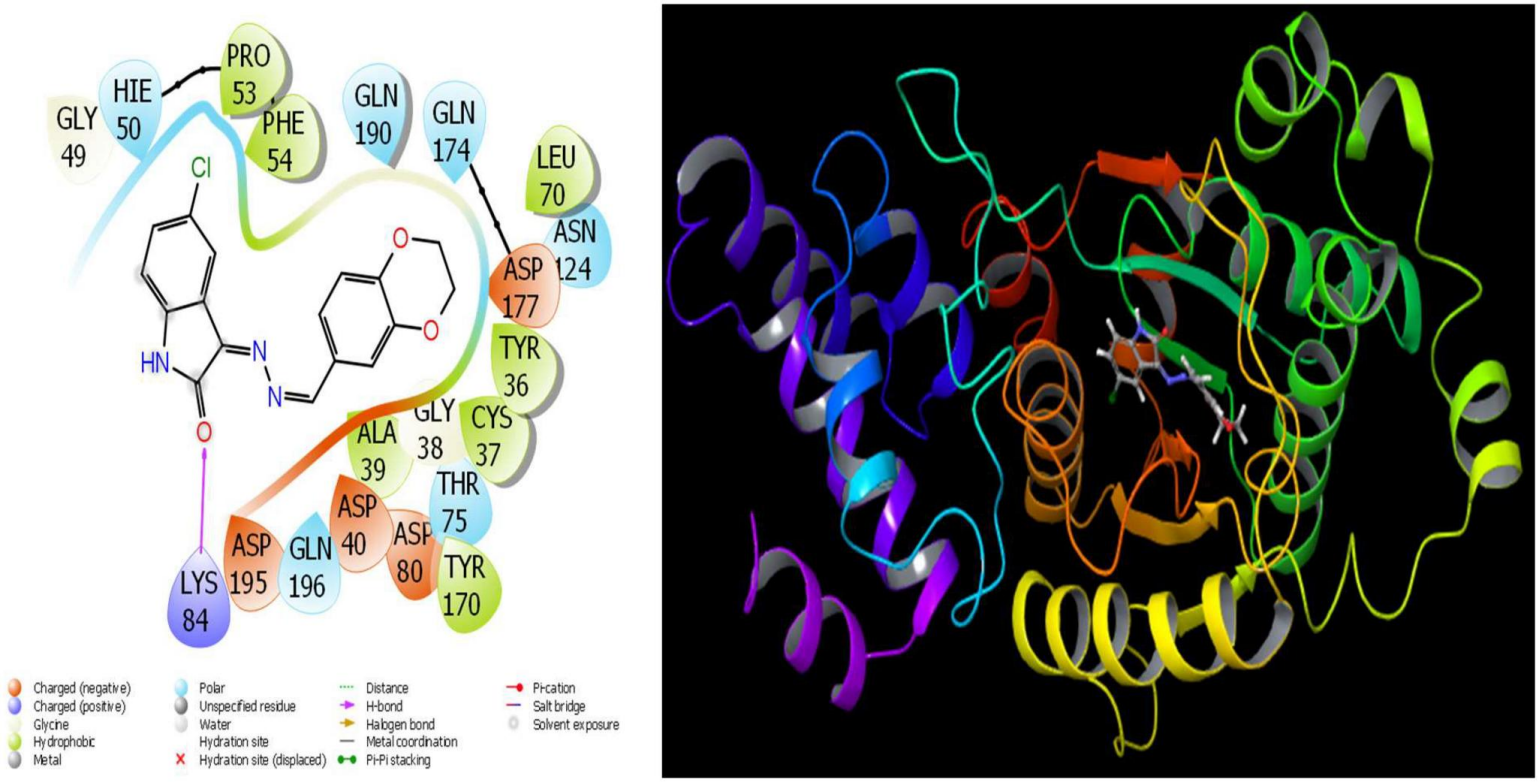
Presentation interactions of molecule 12 with *S. aureus* protein.

The versatility of 1*H*-indole-2,3-dione underlies its wide range of pharmacological and synthetic applications, enabling the creation of diverse intermediates tailored for various chemical purposes. Incorporating pharmacophores into heterocycles has proven to be an effective strategy for generating biologically active analogues. The reaction of free amino groups with various aldehydes produces diverse precursors, with pyridines standing out as compounds possessing a wide range of biological applications^6^^,^^18–20^. For instance, when 3-pyridine carboxaldehyde was reacted with isatin hydrazide under mildly acidic conditions in ethanol as the solvent, it resulted in a moderate yield of 3-((pyridin-3-ylmethylene)-hydrazono)indolin-2-one 3 (see Figure 2). This molecule was reported earlier and found active against lung, MCF7, and hepatocellular cancer cell lines^21^. The identification of the proposed molecule was corroborated by the presence of an exocyclic CH proton signal at 7.63 ppm and the disappearance of the aldehyde signal in the proton spectrum. Acknowledging the constraints of the established methods, we endeavoured to develop a novel and efficient approach. Consequently, employing ethanol in combination with a catalytic quantity of acetic acid yielded a satisfactory yield of 3-(((3-bromobenzo[b]thiophen-2-yl)methylene)-hydrazono)indolin-2-one 4. The ^1^H NMR spectrum of the synthesized molecules displayed the characteristic signal of the exocyclic CH at its designated position, and the aromatic protons exhibited their respective resonance regions.

**Scheme 1.**
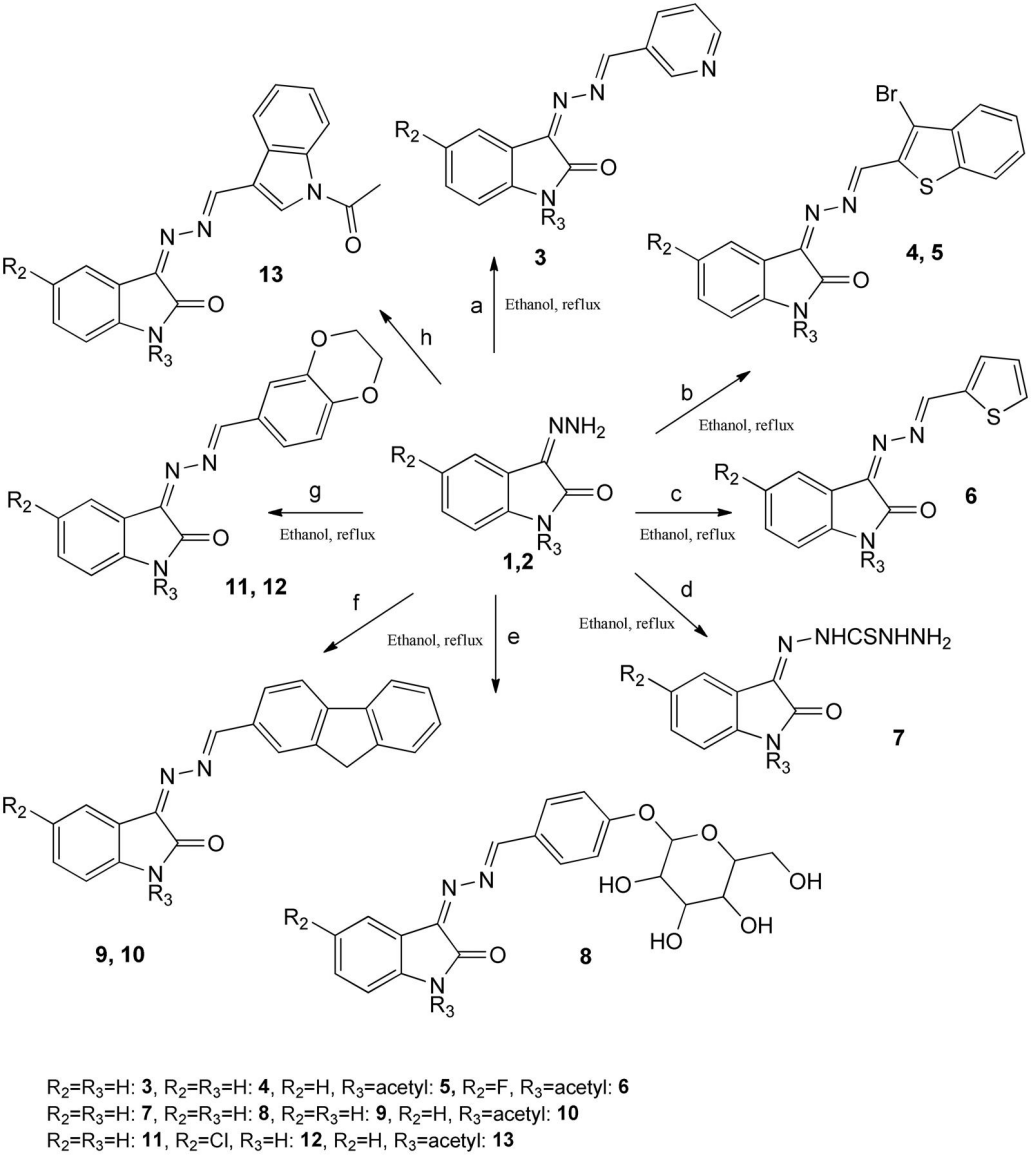
Synthesis of diversified isatin analogues. Ethanol, reflux, **a** = pyridine-3-carboxaldehyde, **b** = 3-bromobenzothiophene-2-carboxaldehyde, **c** = 2-thiophenecarboxaldehyde, **d** = thiosemicarbazide, **e** = 4-(β-d-allopyranosyloxy)benzaldehyde, **f** = fluorene-2-carboxaldehyde, **g** = 1,4-benzodioxan-6-carboxaldehyde, and **h** = indole-3-carbxaldehyde.

Therefore, ethanol is used along with a catalytic amount of acetic acid afforded 3-(((3-bromobenzo[b]thiophen-2-yl)methylene)-hydrazono)indolin-2-one **4** in good yield. In the ^1^H NMR spectrum of synthesized molecules, the signal of exocyclic CH appears at the characteristic position; moreover, aromatic protons appeared in their respective regions. To increase the solubility of the synthesized molecule, **4** was treated with acetic anhydride with a catalytic amount of pyridine afforded 1-acetyl-3-(((3-bromobenzo-[b]thiophen-2-yl)methylene)-hydrazono)indolin-2-one **5** in high yield. Disappearance of NH and the presence of methyl group at *δ* = 2.66 ppm confirms the proposed molecule. The mass and FTIR data are also in accord with the proposed structures of compounds **4** and **5**. To increase the efficacy and to find the most potent analogue, already reported 5-fluoro-3-((thiophen-2-ylmethylene)-hydrazono)indolin-2-one was treated with acetic anhydride afforded 1-acetyl-5-fluoro-3-((thiophen-2-yl)-methylene)hydrazono)indolin-2-one **6** in higher yield. It is reported earlier that incorporating a thiol in heterocycles has led to several analogues possessing interesting biological properties^22^. Therefore, 2-(2-oxoindolin-3-ylidene)-hydrazinethiocarbohydrazide **7** was synthesized by a reaction of **1** with thiocarbohydrazide in ethanol under reflux (Scheme 1). The product was obtained in moderate yield and was confirmed by proton and mass spectrometry. The recent increase in chemotherapeutic agents and the advancement in proteomics have generated several new drug targets, which change the action of chemotherapy at the molecular level. Sugar molecules attached to heterocyclic moiety can enhance the bioavailability and solubility, which is vital for the success of the drug.

Moreover, fluorene and dihydrobenzo[1,4]-dioxane skeletons are well known for their antimicrobial potential. For synthesizing compounds, **8**, **9**, **11**, and **12**, 3-hydrazonoindolin-2-one **3** was treated with 4-(β-d-allopyranosyloxy)-benzaldehyde, fluorene-2-carboxaldehyde, 1,4-benzodioxane-6-carboxaldehyde, and fluorinated analogue of 1,4-benzodioxane-6-carboxaldehyde, respectively in the presence of acetic acid in ethanol (Scheme 1). No aldehyde peak was observed; a new peak corresponds to exocyclic CH proton in the downfield region in all **8**, **9**, **11**, and **12** proton spectrums.

Furthermore, compounds **9** and 3-(((1H-indol-3-yl)methylene)hydrazono)indolin-2-one were treated with an excess of acetic anhydride with few drops of pyridine at room temperature, obtaining **10** and **13**. The primary support for the proposed structures was provided by ^1^H NMR spectra, particularly concerning the protons of the methyl group, which appeared as a singlet at around 2.66 and 2.67 ppm in **10** and **13**, respectively. In molecule **13**, both NH groups were acetylated rendering the molecule highly soluble in most solvents. Mass and FTIR spectrums are also following the proposed structures. 2-Amino-diphenylethanol bearing a chiral centre represents an essential class of substituted isatin. 3-(2-Hydroxy-1,2-diphenylethylimino)indolin-2-one **14** was obtained when chiral aminoethanol was reacted with isatin under reflux in ethanol. In the proton NMR spectrum, two doublets representing two CH groups and the free NH appear as a broad singlet in the downfield region (11.14 ppm), further confirming the postulated structure.

**Scheme 2.**
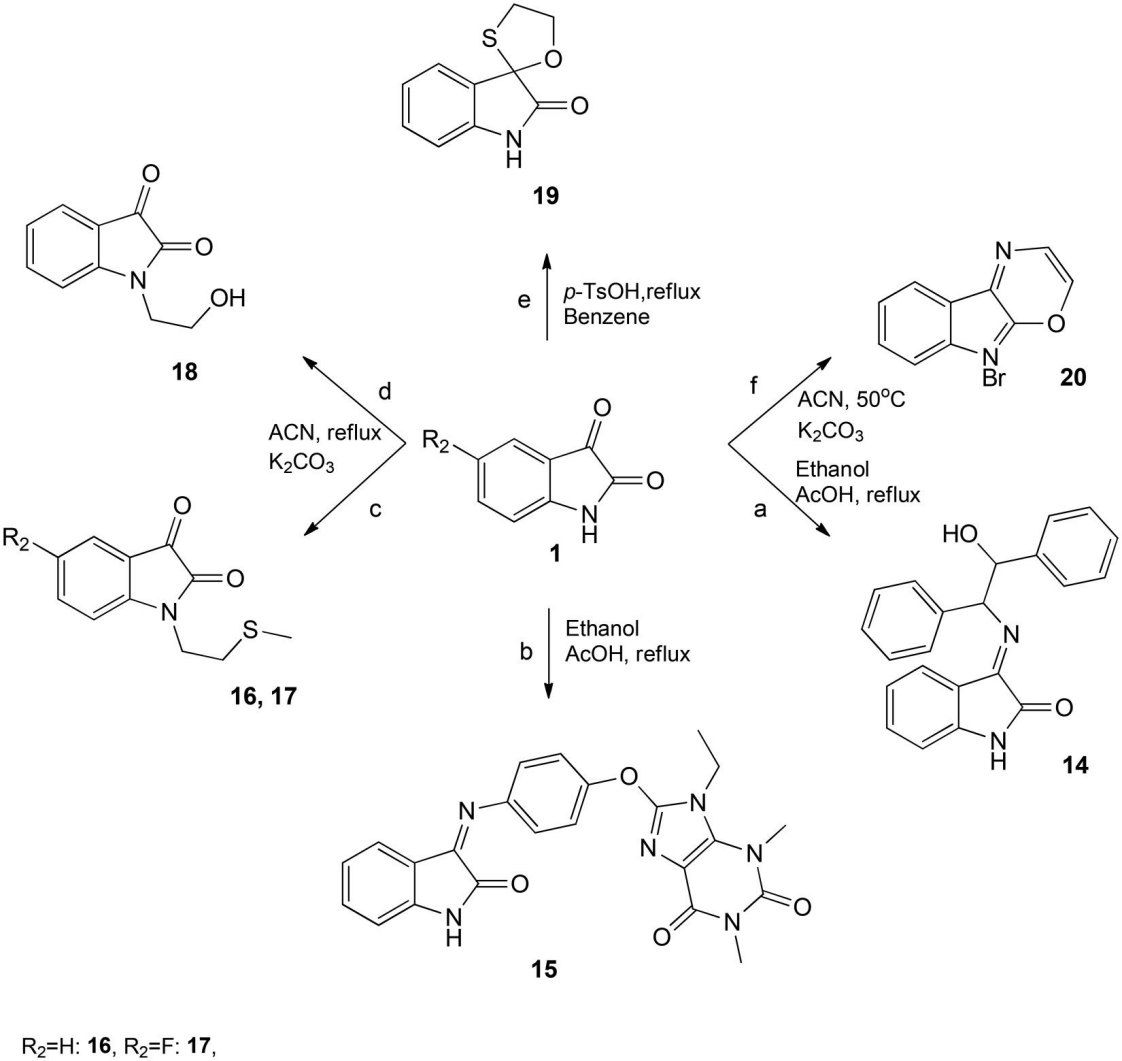
Synthesis of spiro-isatin and imi. **a** = 2-amino-1,2-diphenylethanol, **b** = 8-(4-aminophenoxy)-9-ethyl-1,3-dimethyl-1H-purine-2,6(3H,9H)-dione, **c** = 2-chloroethylmethyl sulphide, **d** = bromoethanol, and **e** = 2-mercaptoethanol.

Moreover, the purine heterocyclic system, the parent ring in various analogues of biological importance, is considered an essential natural ring system^23^. In this work, the construction of purine heterocyclic system was carried out by a reaction of isatin **1** with 8-(4-aminophenoxy)-9-ethyl-1,3-dimethyl-1*H*-purine-2,6(3*H*,9*H*)-dione rendered **15** in moderate yield (Scheme 2). The product is confirmed by one- and two-dimensional NMR and mass spectroscopy.

**Figure 3.**
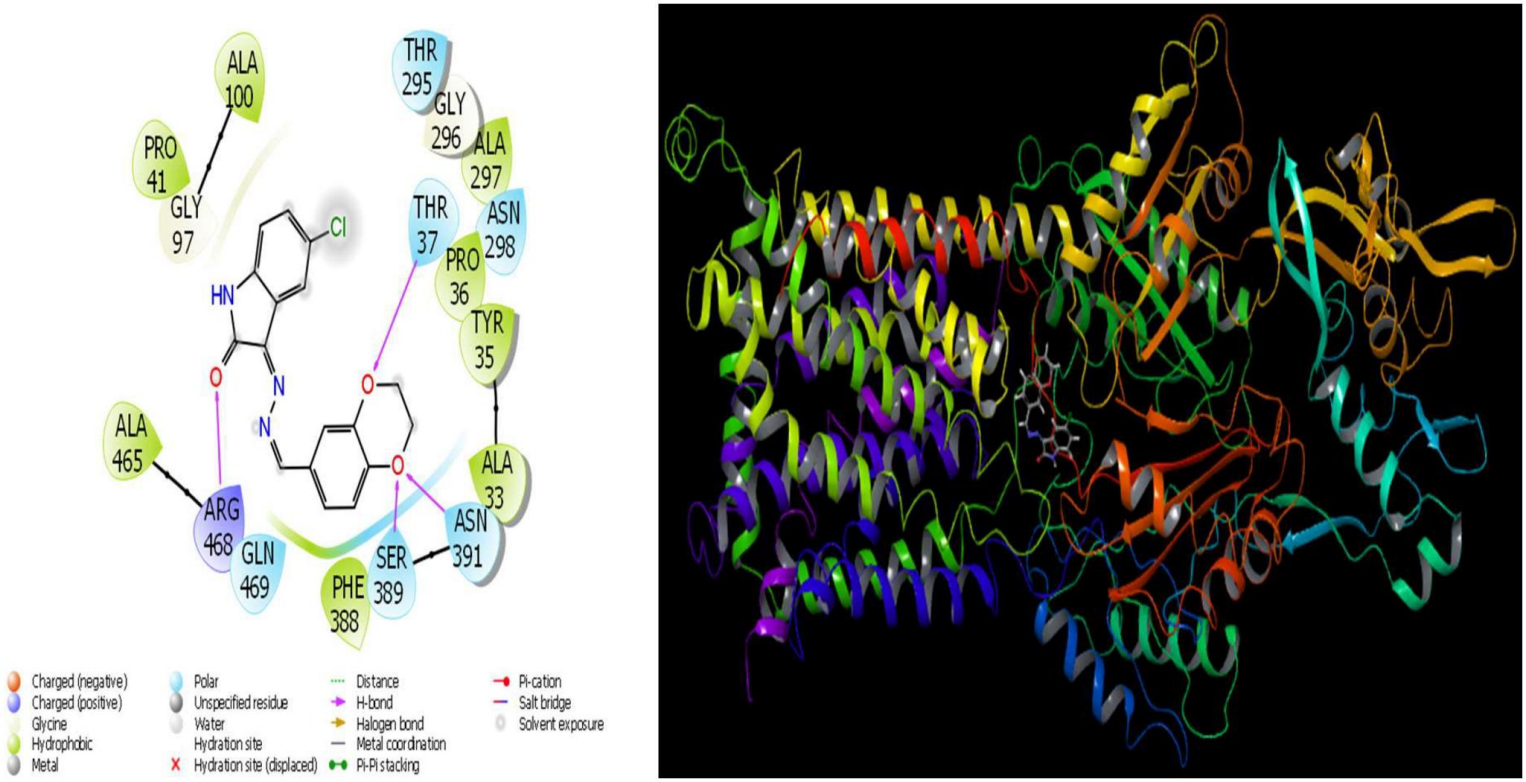
Presentation interactions of molecule 12 with *Escherichia coli.*

Alkylating the nitrogen atom within isatin leads to the generation of several biologically significant derivatives. These derivatives serve as versatile synthetic intermediates capable of producing a diverse range of promising pharmaceutical compounds. While previous attempts at synthesizing these derivatives have been documented, they have encountered significant challenges due to the demanding reaction conditions and the formation of various unwanted byproducts, rendering the isolation of the desired molecules unfeasible^24^. In this study, *N*-alkylation of isatin was executed by subjecting 2-chloroethylmethyl sulphide and bromoethanol to a reaction in the presence of potassium carbonate, conducted under reflux conditions in acetonitrile. The resulting mixture yielded products **16**, **17**, and **18** in substantial yields, and these were subsequently precipitated upon addition to ice water (refer to Figure 3). Confirmation of the proposed molecules was obtained through the disappearance of the NH proton and the emergence of thiomethyl protons, corresponding to SCH_3_ in the upfield region (approximately 2.3 ppm) in the proton NMR spectrum. Before undergoing spectroscopic characterization, all synthesized compounds were meticulously washed with water and cold ethanol.

Furthermore, spiro derivatives of isatin have been found to produce main pharmacological activity, especially in natural product chemistry^25^. Various studies have been reported involving the C-3 position of isatin in synthesizing spiro analogues like spiroxindole. The major drawback of the written protocol for synthesizing oxathiino[2,3-b]indole **19** is the low yield and formation of side products. In the current paper, we developed a facile route to synthesize 3,4*a*,5,9*b*-tetrahydro-2*H*-[1,4]oxathiino[2,3-*b*]indole **19** in our search for the synthesis of a diverse range of biologically active heterocyclic compounds. *Para* toluenesulfonic acid (*p*-TsOH) is used as an active catalyst in benzene to synthesize spirthioindole in excellent yields. In the reaction of isatin with bromoethyl amine, acetonitrile was used with K_2_CO_3_. The product was obtained after prolonged heating at 50 °C while no side products were observed. The synthesized compounds **19** and **20** were well characterised by spectral data. In ^1^H NMR spectra, the appearance of CH_2_ at around 3–4 ppm in **19** and two doublets that correspond to CH attached to N and O in the downfield region in **20** are proof for the proposed structures (Scheme 2). The IR spectra of all the synthesized compounds also showed spectral bands in their respective regions.

### Biology

#### Anticancer evaluation

All the synthesized compounds were subjected to an *in vitro* antiproliferative screening against 60 different cancer cell lines at the National Cancer Institute (NCI)^26^. The mean percentages of growth inhibition (GI %) of the tested compounds are shown in Table 1. Human cell lines that were used in the current paper are derived from leukaemia, lung, colon, CNS, melanoma, ovarian, renal, prostate, and breast cancers. Some of the synthesized compounds showed excellent anti-proliferative activity when treated with a concentration of 10 mM as presented in Table 2. The inhibitory activity of compounds in the series indicates some sort of inhibition with the enzyme albeit weak. Moreover, it is imperative that the activity is primarily selective to those compounds featuring privileged groups attached to isatin moiety^27^. For example, molecules **3** (34.14%), **4** (34.38%), **19** (37.09%), **6** (41.34%), and **16** (43.82%) showed excellent activity against renal cancer cell line UO-31 (Table 1). This is assumed to be due to the biological activity asserted by the attached group, which plays a major role in reducing cytotoxicity.

**Table 1.** Growth inhibition of selected compounds.

Sample codes	% GI leukaemia	% GI CNS	% GI renal cancer
	*K-562*	*SNB-75*	*UO-31*
**3**	0	0	34.14
**4**	0	0	34.38
**6**	35.92	35.5	41.34
**15**	0	34.42	0
**16**	0	0	43.82
**19**	0	36.56	37.09

**Table 2.** Activity comparison against various cell lines.

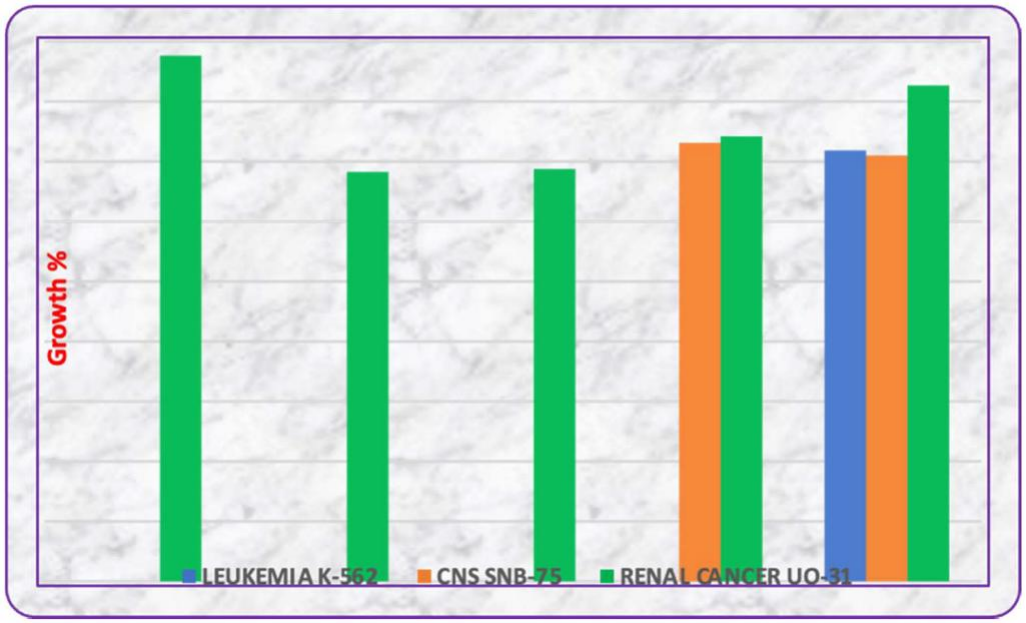

The Schiff bases of isatin **3**, **4**, **6**, and **15** enhance inhibition compared with that of fluorene and benzodioxane. This could be due to the biological activity associated with thiophene and benzothiazole moiety, while the benzothiazole bearing a bromo group also plays a role in increased biological activity. It was anticipated that the presence of a purine nucleus enhances the inhibition considerably, as in the case of **15**. Moreover, the impact of the isatin group in the overall activity is also considered. Compound **6** is found to be the most potent molecule showing inhibition against leukaemia (35.92%), central nervous system (35.5%), and renal cancer (41.34%) cell lines. Compound **9** was most active on the SNB-75 CNS cell line with a growth inhibition of 36.56% and the UO-31 renal cancer cell line with a growth inhibition of 37.09%. This activity is due to the spirothio indole nucleus, which is proven to be active against various cell lines^28^.

#### Antimicrobial activity

The synthesized compounds underwent testing against five bacterial and two fungal strains, revealing that some of these compounds demonstrated activity against fungi, with the most notable candidates being compounds **3** and **16** (refer to Table 2). Compound **16**, in particular, displayed exceptional cytotoxicity against *Candida albicans* and *Cryptococcus neoformans* at an initial concentration of 32 μg/ml, as detailed in Table 3. Furthermore, compound **3** exhibited moderate activity against *Staphylococcus aureus*, a gram-positive bacterium^29^. For the compounds with relatively weak inhibitory effects identified in this study, it would be imperative to consider further structural modifications, such as extending the structure or incorporating bioactive or related groups, to enhance their inhibitory potency.

**Table 3.** Antimicrobial data of selected molecule.

Sample code	Sa	Ec	Kp	Pa	Ab	Ca	Cn
**3**	49.97	6.32	12.5	7.23	9.28	3.54	−5.2
**9**	11.69	−14.92	11	17.99	5.91	12.93	−23.96
**11**	10.22	4.68	19.03	14.01	20.06	6.51	−0.62
**12**	6.3	−15.06	1.88	7.27	10.37	2.1	10.2
**15**	16.65	1.1	13.69	7.86	10.91	4.76	6.65
**16**	23.33	0.34	8.76	13.2	3.47	97.65	95.05

Sa: *Staphylococcus aureus*; EC: *Escherichia coli*; Kp: *Klebsiella pneumoniae*; Pa: *Pseudomonas aeruginosa*; Ab: *Acinetobacter baumannii*; Ca: *Candida albicans*; Cn: *Cryptococcus neoformans var. grubii*.

#### Structure–activity relationship

According to the biological activity data and the structural variations between the molecules, the structural modification of indolin to *N*-acetylindolin along with thiophene moiety led to improvement in cytotoxicity results that may be explained by increasing hydrogen bond formation in **6**. Regarding groups present at the benzothiophene ring, **4** exhibited higher activity within the same type of molecules and had more contribution in the cytotoxicity than the other groups. The electron-withdrawing group on benzothiophene exhibited moderate to significant influence on antitumor activity. Meanwhile, in **3**, pyridine displayed higher cytotoxicity among other groups like benzylidine and fluorene, suggesting that π-electron delocalisation at the pyridine ring has a higher effect as compared with others.

Regarding groups protecting the indolinedione ring, methylthio in **16** exhibited higher activities as compared to the hydroxyethyl group in **18**, indicating that the thiol group has more contribution to the cytotoxicity than the alcohol group. Compound **16** also showed excellent antifungal activity against *Candida albicans* and *Cryptococcus neoformans*, which confirms the strong effect exerted by the methylthiol group on biological activity. The same effect has been observed in **19,** which exhibited better cytotoxicity as compared to **20**, due to oxothiino indole moiety. This study may provide useful information for further design of isatin hybrids having better antitumor activity.

## Molecular modelling

In general, molecular docking calculations are carried out to support experimental activities, to design more stable and more active molecules, and to detect active sites of molecules. Molecular modelling is an important method that examines the interactions of molecules with proteins through molecular docking calculations. This method determines the activity of molecules against proteins and the interaction between them, and as this interaction increases, the activity of the molecules increases. In this study, the microorganism used is Sa: *Staphylococcus aureus*, EC: *Escherichia coli*, Kp: *Klebsiella pneumonia*, Pa: *Pseudomonas aeruginosa*, Ab: *Acinetobacter baumannii*, Ca: *Candida albicans*, and Cn: *Cryptococcus neoformans var. grubii* against synthesised molecules. The theoretical calculation results of the four bacteria that showed the most agreement as a result of the theoretical calculation are given in the study. It is observed that hydrogen bonds, polar and hydrophobic interactions, π–π, and halogen bond interactions occur between molecules and proteins. The interactions that occur are given in Figures 2 and 3. As a result of the calculations, many parameters have been calculated, and each calculated parameter gives information about the different properties of the molecules. When these parameters are examined, the first parameter that determines the activities of the molecules is the docking score parameter. Moreover, glide bond, glide van der Waals energy, and Glide ecoul parameters give numerical values of interactions between molecules and proteins.

**Figure 4.**
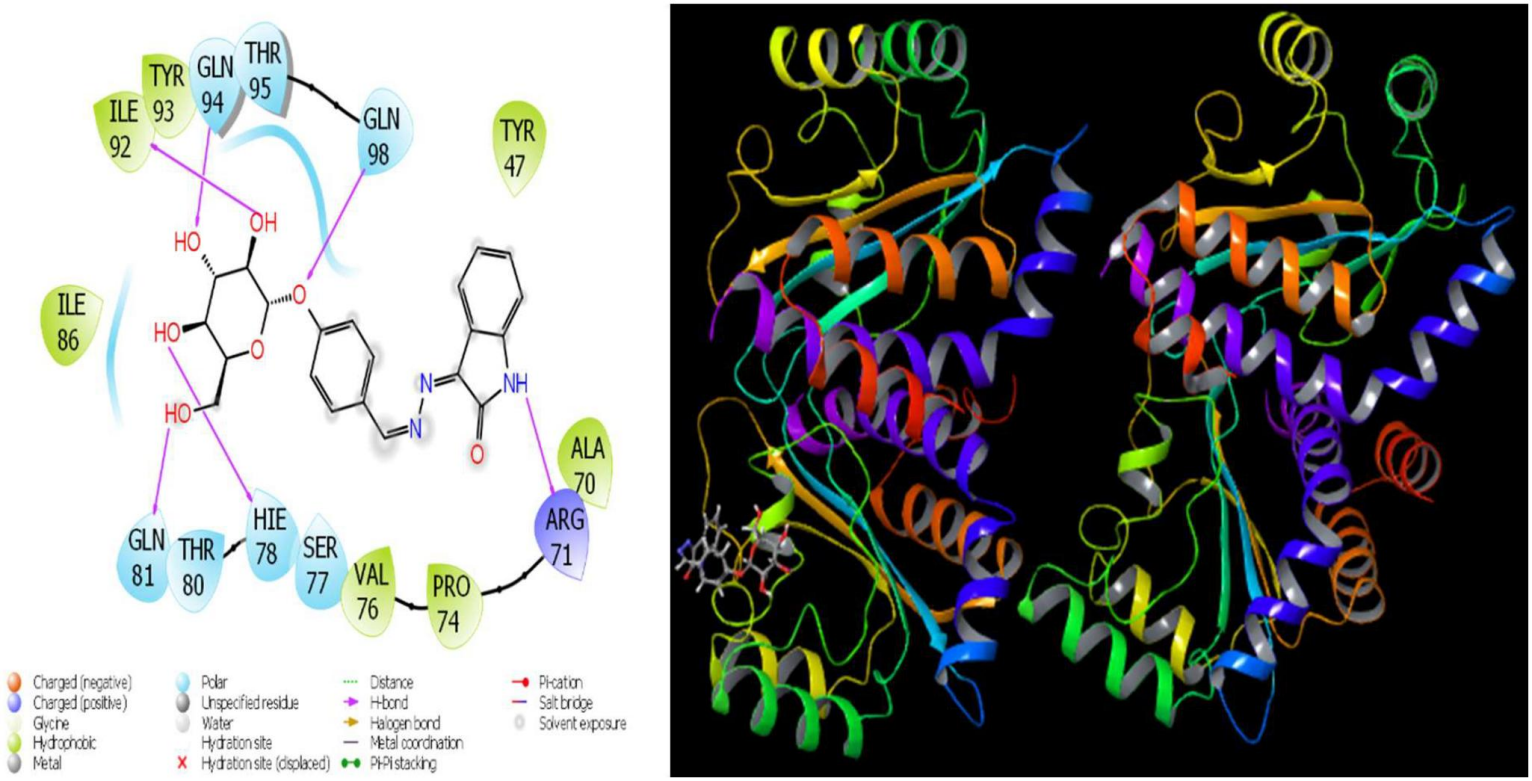
Presentation interactions of molecule 8 with *Pseudomonas aeruginosa* protein.

**Figure 5.**
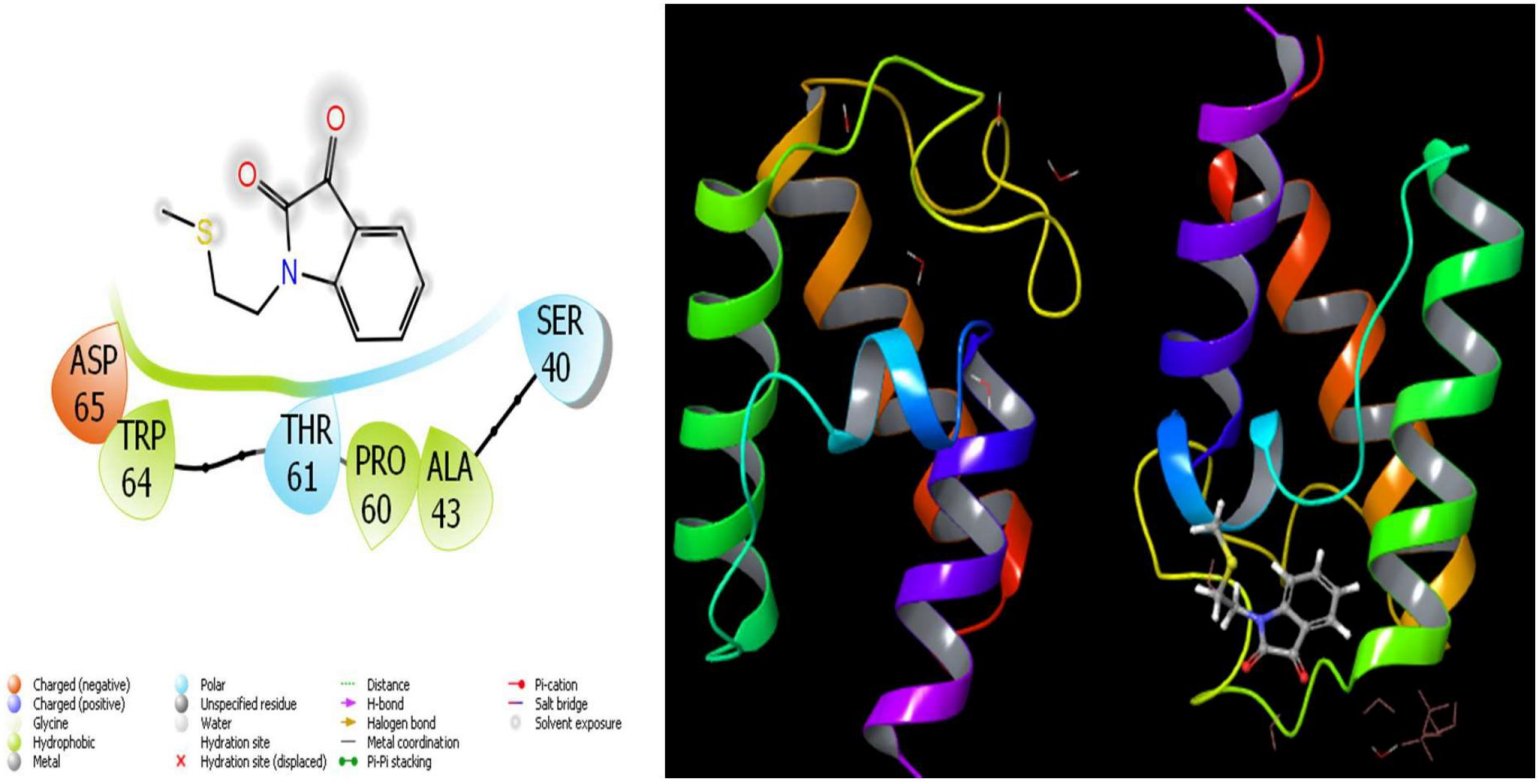
Presentation interactions of molecule 6 with *Acinetobacter baumannii* protein.

The parameters that give information about the interaction pose between molecules and proteins are glide model, glide energy, glide internal, and glide posenum. In the current paper, the interaction between molecule **12** and the *Staphylococcus aureus* protein is given in Figure 2, and it is seen that hydrogen bonding occurs between the oxygen atom attached to the carbonyl carbon in molecule **12** and the LYS 84 protein in this interaction. The interaction between **12** and *Escherichia coli* protein is given in Figure 3, and it is evident that hydrogen bonding occurs between the oxygen atom attached to the carbonyl carbon in molecule **12** and the ARG 468 protein in this interaction. It is also observed that one of the oxygen atoms in the 2,3-dihydrobenzo[*b*][1,4]dioxane ring in molecule **12** forms a hydrogen bond with the THR 37 protein, and the other oxygen atom forms a hydrogen bond with the SER 389 and ASN 391 protein. Furthermore, the interaction between molecule **8** and the *Pseudomonas aeruginosa* protein is shown in Figure 4 and it is observed that the hydrogen bonding occurs between the hydrogen atom attached to the amine in the indolin-2-one ring in molecule **8** and the ARG 71 protein. The hydroxy groups in the 2-(hydroxymethyl)tetrahydro-2*H*-pyran-3,4,5-triol ring in molecule **8** appear to form hydrogen bonds with GLN 94, ILE 92, HIE 78, and GLN 81 proteins. It is envisaged that hydrogen bonding occurs between the oxygen atom in the centre of molecule **8** and the GLN 98 protein. Finally, in Figure 5, it is seen that polar and hydrophobic interactions occur between molecule **6** and the protein of *Acinetobacter baumannii*.

ADME/T analysis (absorption, distribution, metabolism, excretion, and toxicity) was performed to examine the effects and responses of these studied molecules in human metabolism. With this analysis, the absorption of molecules by human metabolism, distribution in human metabolism, excretion from metabolism, and finally toxicity values in metabolism are calculated^30^^,^^31^. The parameters calculated here are examined for chemical as well as biological properties of molecules. As a result of these ADME/T calculations, the calculated parameters of the molecules are given in Table 4. Although the calculated chemical and biological parameters of the molecules found in this study are within the desired values for use in human metabolism as a drug, the numerical values of the rule of five (ROF)^32^^,^^33^ and rule of three (ROT)^34^ parameters are two important parameters considered for their use as drugs. Although the ROF parameter is generally known as the number of violations of *Lipinski’s rule of five*, it is known to contain four parameters. On the other hand, the ROT parameter is known as several numbers of violations of *Jorgensen’s rule of three*.

**Table 4.** ADME properties of molecule.

	3	4	5	6	7	8	9	10	11	Reference range
mol_MW	426	384	426	315	235	427	337	379	307	130–725
dipole (D)	6.4	4.0	6.4	5.1	4.7	3.8	3.4	5.3	4.0	1.0–12.5
SASA	656	592	656	575	475	717	650	718	562	300–1000
FOSA	100	18	100	106	20	148	78	162	147	0–750
FISA	98	103	98	105	238	255	102	101	102	7–330
PISA	368	382	368	288	182	313	469	455	313	0–450
WPSA	90	90	90	76	35	0	0	0	0	0–175
volume (A^3^)	1145	1013	1145	976	764	1276	1123	1257	969	500–2000
donorHB	0	1	0	0	4	5	1	0	1	0–6
accptHB	5.5	5	5.5	5.5	6	14.2	5	5.5	6.5	2.0–20.0
glob (Sphere = 1)	0.8	0.8	0.8	0.8	0.9	0.8	0.8	0.8	0.8	0.75–0.95
QPpolrz (A^3^)	41.3	36.1	41.3	33.7	23.0	41.5	40.0	45.3	33.7	13.0–70.0
QPlogPC16	12.8	11.8	12.8	9.9	9.3	15.3	12.9	13.9	10.3	4.0–18.0
QPlogPoct	17.5	16.7	17.5	14.6	17.2	29.3	17.6	18.3	16.2	8.0–35.0
QPlogPw	9.3	10.2	9.3	8.6	14.4	24.5	10.5	9.5	11.1	4.0–45.0
QPlogPo/w	4.2	3.6	4.2	2.9	−0.5	−0.2	4.1	4.6	2.3	−2.0 to 6.5
QPlogS	−5.7	−5.2	−5.7	−4.1	−1.1	−3.1	−5.5	−6.1	−3.7	−6.5 to 0.5
CIQPlogS	−6.7	−6.1	−6.7	−4.1	−1.2	−3.3	−5.3	−5.8	−3.9	−6.5 to 0.5
QPlogHERG	−6.4	−6.2	−6.4	−5.7	−5.5	−6.4	−6.9	−7.2	−5.6	^a^
QPPCaco (nm/s)	1163	1047	1163	999	14	38	1070	1085	1070	^b^
QPlogBB	−0.4	−0.4	−0.4	−0.5	−1.4	−2.7	−0.8	−0.8	−0.6	−3.0 to 1.2
QPPMDCK (nm/s)	1806	1612	1806	1291	8	14	532	540	532	^b^
QPlogKp	−1.7	−1.8	−1.7	−2.2	−6.8	−4.2	−1.3	−1.3	−2.0	Kp in cm/h
IP (eV)	8.7	8.6	8.7	8.8	8.3	8.7	8.7	8.8	8.7	7.9–10.5
EA (eV)	1.6	1.5	1.6	1.5	1.1	1.0	1.0	1.1	1.0	−0.9 to 1.7
#metab	2	2	2	2	1	5	1	1	1	1–8
QPlogKhsa	0.3	0.3	0.3	−0.1	−0.6	−0.9	0.5	0.6	−0.1	−1.5 to 1.5
Human oral absorption	3	3	3	3	2	2	3	3	3	–
Percent human oral absorption	100	100	100	100	44	54	100	100	95	^c^
PSA	84	70	84	83	134	168	71	85	87	7–200
RuleOfFive	0	0	0	0	0	0	0	0	0	Maximum is 4
RuleOfThree	0	0	0	0	1	0	0	1	0	Maximum is 3
Jm	0.0	0.0	0.0	0.2	0.0	0.0	0.1	0.0	0.6	–

	12	13	14	15	16	17	18	19	20	Reference range
mol_MW	342	330	342	432	221	239	191	207	–	130–725
dipole (D)	1.7	3.5	4.8	9.0	5.5	4.9	4.9	4.7	–	1.0–12.5
SASA	587	605	607	735	461	470	402	406	–	300–1000
FOSA	147	100	7	180	155	155	78	111	–	0–750
FISA	102	141	107	226	102	102	158	97	–	7–330
PISA	266	364	494	329	166	128	166	169	–	0–450
WPSA	72	0	0	0	38	84	0	30	–	0–175
volume (A^3^)	1015	1067	1094	1296	756	771	650	663	–	500–2000
donorHB	1	1	2	2	0	0	1	1	–	0–6
accptHB	6.5	8	5.2	8.5	5.5	5.5	6.7	4.7	–	2.0–20.0
glob (Sphere = 1)	0.8	0.8	0.8	0.8	0.9	0.9	0.9	0.9	–	0.75–0.95
QPpolrz (A^3^)	35.1	38.1	38.5	46.9	23.8	24.0	19.5	20.1	–	13.0–70.0
QPlogPC16	10.9	11.9	13.1	14.7	7.3	6.9	6.8	6.8	–	4.0–18.0
QPlogPoct	16.7	18.7	18.9	24.5	11.0	11.2	11.8	10.7	–	8.0–35.0
QPlogPw	10.9	13.3	12.0	15.9	7.4	7.2	10.3	7.9	–	4.0–45.0
QPlogPo/w	2.8	2.1	3.7	2.6	1.2	1.4	−0.2	1.2	–	−2.0 to 6.5
QPlogS	−4.5	−3.9	−4.4	−6.0	−1.8	−2.0	−1.1	−1.9	–	−6.5 to 0.5
CIQPlogS	−4.6	−3.8	−5.3	−6.0	−1.8	−2.1	−1.2	−2.2	–	−6.5 to 0.5
QPlogHERG	−5.5	−5.9	−6.4	−6.6	−4.4	−4.3	−3.9	−3.9	–	^a^
QPPCaco (nm/s)	1070	461	963	72	1075	1075	312	1202	–	^b^
QPlogBB	−0.4	−1.0	−0.8	−2.1	−0.4	−0.3	−0.9	−0.3	–	−3.0 to 1.2
QPPMDCK (nm/s)	1320	214	475	29	864	1551	141	879	–	^b^
QPlogKp	−2.2	−2.5	−1.2	−4.2	−2.5	−2.7	−3.6	−2.4	–	Kp in cm/h
IP (ev)	8.8	8.5	9.1	8.1	9.0	9.1	9.4	9.1	–	7.9–10.5
EA (eV)	1.1	1.2	0.9	1.0	1.1	1.3	1.0	1.0	–	−0.9 to 1.7
#metab	1	1	4	1	0	0	1	1	–	1–8
QPlogKhsa	0.0	−0.2	0.3	0.3	−0.8	−0.7	−0.9	−0.5	–	−1.5 to 1.5
Human oral absorption	3	3	3	3	3	3	2	3	–	–
Percent human oral absorption	100	87	100	75	88	90	70	89	–	^c^
PSA	87	97	72	144	60	60	83	56	–	7–200
RuleOfFive	0	0	0	0	0	0	0	0	–	Maximum is 4
RuleOfThree	0	0	0	1	0	0	0	0	–	Maximum is 3
Jm	0.1	0.1	0.9	0.0	11.7	4.0	4.1	11.2	–	–

^a^ Corcern below –5.

^b^ <25 is poor and >500 is great.

^c^ <25% is poor and >80% is high.

### Docking with 1A06 protein

**Figure 6.**
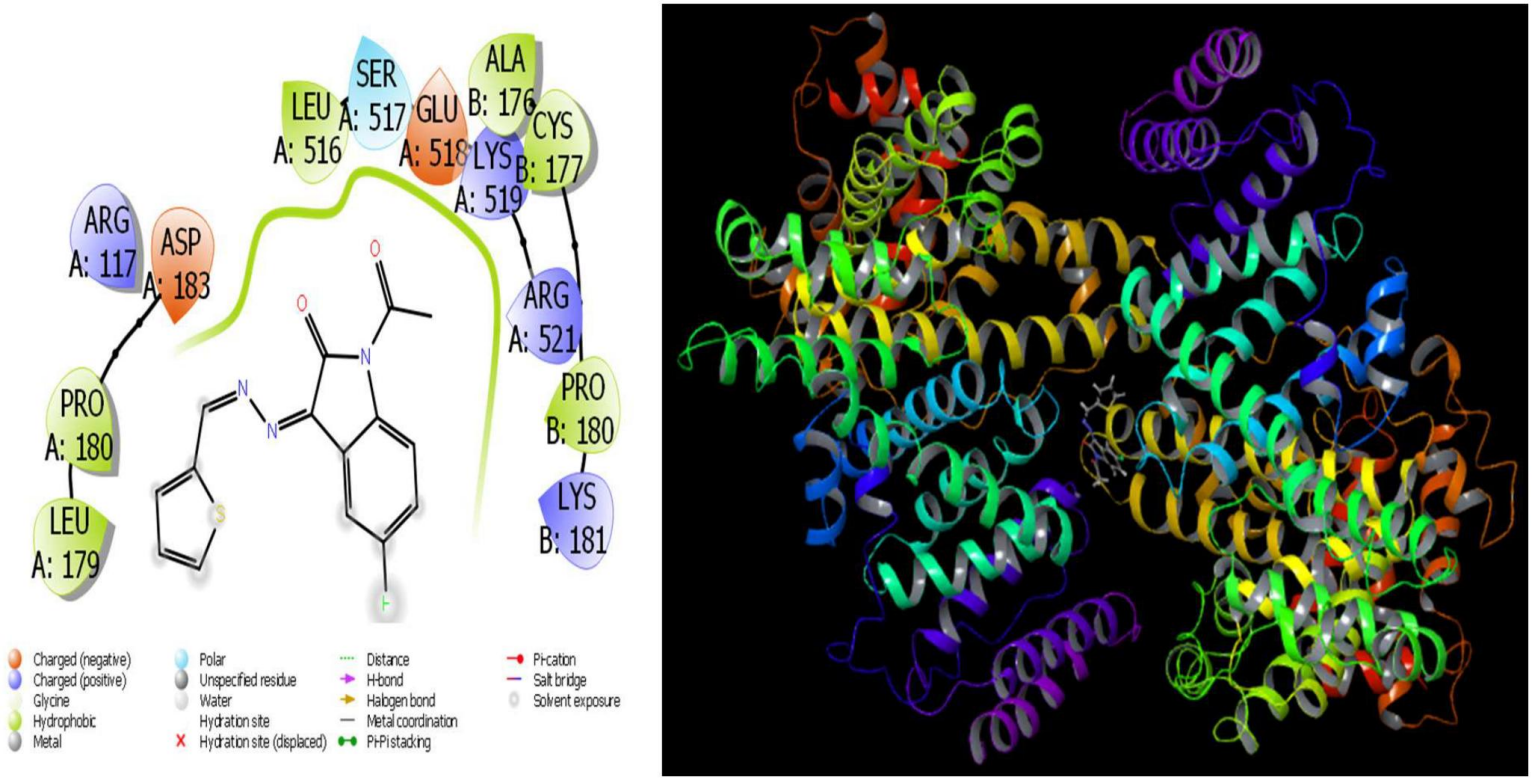
Presentation interactions of molecule 6 with 1A06 protein.

**Figure 7.**
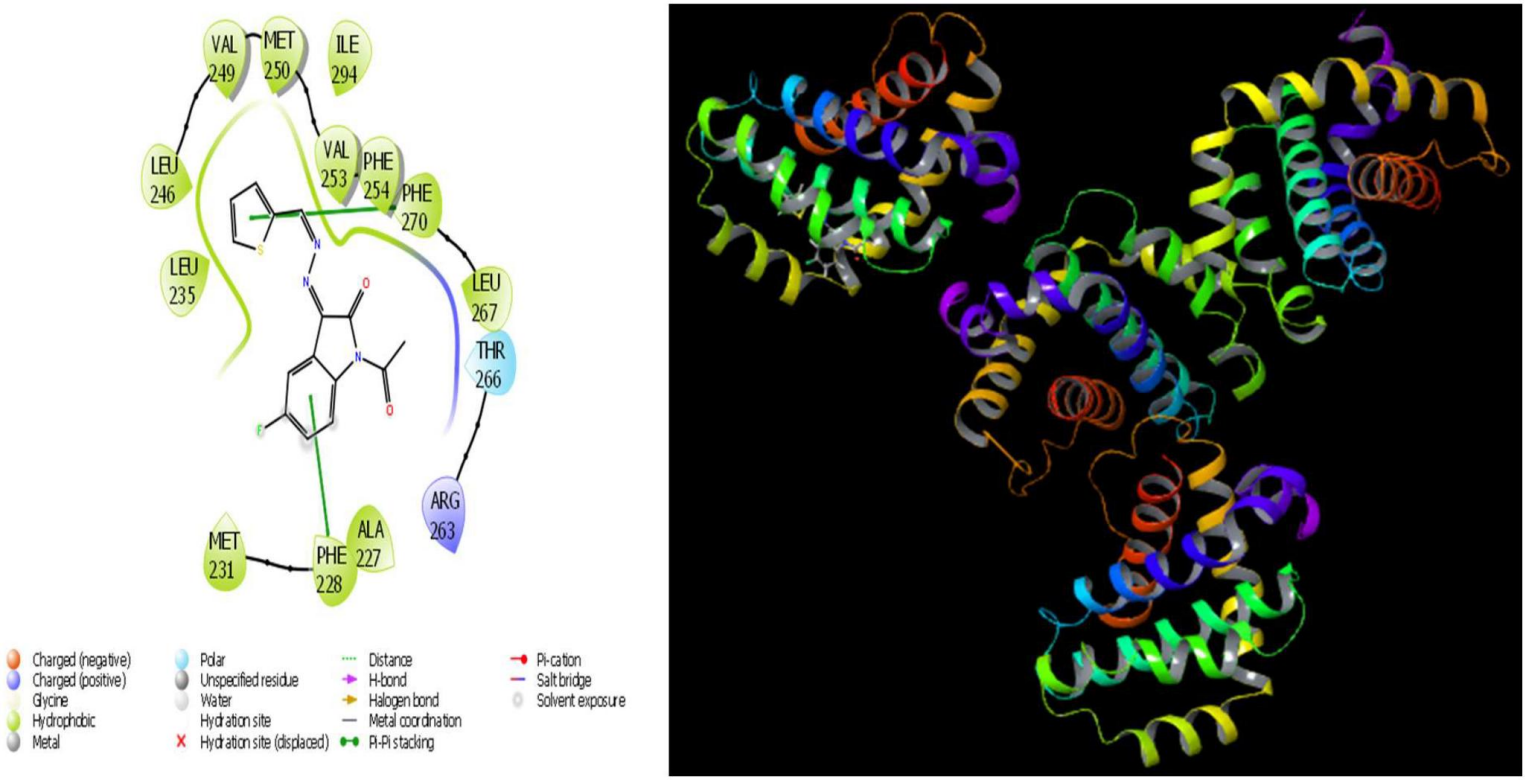
Presentation interactions of molecule 6 with 6BW2.

In the current paper, the interaction between molecule **6** and the 1A06 protein is given in Figure 6, and it is seen that hydrogen bonding occurs between the imine moiety in molecule **6** and the PHE 254 protein in this interaction. Moreover, the interaction between molecule **6** and 1A06 *protein* is given in Figure 6 and it is evident that hydrogen bonding occurs between the exocyclic nitrogen double bond in molecule **6** and the PHE 270 protein in this interaction. It is also observed that the phenyl ring in molecule **6** forms a hydrogen bond with the PHE 228 protein and MET 231 protein as evident in Figure 7.

## Materials and methods

All solvents and reagents were purchased from Aldrich Chemical Co. (St. Louis, MO) and were used as obtained. The reactions were carried out under air atmosphere unless stated otherwise. IR spectra were recorded with a Bruker Alpha spectrometer (Billerica, MA).^1^H- and ^13^C NMR spectra were recorded on a Bruker instrument at 500 and 700 MHz for ^1^H and 176 MHz for ^13^C at 295 K in DMSO-*d*_6_ and CDCl_3_. The mass spectrometric experiments were carried out on a Jeol JMS-700 mass spectrometer (Akishima, Japan). Thin-layer chromatography (TLC) was performed on a fluorescent aluminium backed silica gel HF^254^ plates (Merck, Kenilworth, NJ) and was viewed under UV 254 and 265 lights and charring with EtOH/H_2_SO_4_. Merck silica gel 60 (230–400 mesh) was used for column chromatography separations.

### General procedure for the synthesis of compounds 4, 6, 8, 9, and 11

A mixture containing the respective isatin hydrazide (1.1 mmol) and appropriate aldehyde (1.0 mmol) with catalytic amount of acetic acid was heated under reflux and stirring for 3 h in dry ethanol (20 ml). After completion of the reaction, as indicated by TLC, the reaction mixture was poured onto crushed ice and the solid separated was filtered, washed with ice-cold water several times (50 ml) and subsequently dried to afford pure product.

### General procedure for the synthesis of compound 7

A mixture containing the isatin (1 mmol) and thiosemicarbazide (1.1 mmol) with catalytic amount of acetic acid was heated under reflux and stirring for 2 h in dry ethanol (20 ml). After completion of the reaction, the mixture was poured onto crushed ice and the solid separated was filtered, washed with ice-cold water several times (50 ml) and subsequently dried to afford pure product. The obtained product was dissolved in excess of acetic anhydride (25 ml) with few drops of pyridine and stirred overnight at room temperature. The solid obtained after adding ice was filtered and washed with water and dried to afford pure product.

### General procedure for the synthesis of compounds 5, 10, 12, and 13

To a vigorously stirred solution of respective imines (1.0 mmol) in acetic anhydride (50 ml) was added few drops of pyridine. The resulting mixture was stirred overnight for completion of the reaction and the progress of the reaction was monitored by TLC. The solvent was evaporated to afford the crude product, which was poured onto crushed ice and the separated solid was filtered, washed several times with cold ethanol (25 ml) to obtain pure product.

### General procedure for the synthesis of compounds 16, 17, 18, and 20

A mixture containing the respective isatin hydrazide (1.1 mmol), 2-chloroethylmethyl sulphide, and/or bromoethanol (1.5 mmol) respectively was refluxed in acetonitrile (20 ml) in the presence of K_2_CO_2_ (2.0 mmol) for 3 h. The mixture was then stirred at room temperature for completion of the reaction, as indicated by TLC. The product obtained was poured onto crushed ice and the solid separated was filtered, washed with ice-cold water several times (50 ml) and subsequently dried to afford pure product.

### General procedure for the synthesis of compound 19

To a vigorously stirred solution of isatin (1.1 mmol) in benzene (50 ml) was added mercaptoethanol (2 mmol) and *p*-TsOH (0.01 mmol). The resulting mixture was stirred at room temperature for 1 h and then refluxed for 5 h. The progress of the reaction was monitored by TLC. After completion of the reaction, the product was evaporated and purified by column chromatography (1:2 hexane–ethylacetate).

#### *3-(((3-Bromobenzo[b]thiophen-2-yl)methylene)hydrazono)indolin-2-one (*4*)*

Yield 68%, yellowish brown solid, m.p.: 244–247 °C. IR: 1715 and 3100 cm^−1^: ^1^H NMR (700.170 MHz, DMSO-*d*_6_): *δ* = 6.93 (d, 1H, *J* = 7 Hz, Ph), 7.08 (s, 1H, Ph), 7.45 (s, 1H, Ph), 7.62, 7.65 (m, 2H, Ph), 7.93 (d, 2H, *J* = 7 Hz, Ph), 8.19 (s, 1H, CH), 8.93 (s, 1H, Ph), 10.94 (s, 1H, NH). ^13^C NMR (DMSO-*d*_6_) *δ* = 111.5, 116.4, 124.1, 124.2, 124.5, 126.8, 127.0, 129.1, 129.5, 130.1, 133.9, 138.2, 145.9, 152.0, 154.9, 164.8, and 185.7.

MS (ESI, 70 eV): *m/z* 383 (MH)^+^. Anal. Calcd. for C_17_H_10_BrN_3_OS (382.97).

#### *1-Acetyl-3-(((3-bromobenzo[b]thiophen-2-yl)methylene)hydrazono)indolin-2-one (*5*)*

Yield 88%, light yellow solid, m.p.: 171–174 °C. IR: 1710 and 3070 cm^−1^: ^1^H NMR (700.170 MHz, DMSO-*d*_6_): 2.67 (s, 3H, CH_3_), 7.39 (s, 1H, Ph), 7.64–7.68 (m, 3H, Ph), 7.96 (d, 1H, *J* = 7 Hz, Ph), 8.21 (s, 1H, CH), 8.28 (dd, 2H, *J* = 4 Hz, Ph), and 8.98 (s, 1H, Ph). ^13^C NMR (DMSO-*d*_6_) *δ* = 26.9, 116.9, 117.9 124.1, 124.4, 125.9, 126.9, 129, 129.3, 133.8 (2×), 134.5, 138.2, 139.4, 143.4, 149.9, 155.6, 163.7, and 170.6.

MS (ESI, 70 eV): *m/z* 425 (MH)^+^. Anal. Calcd. for C_19_H_12_BrN_3_O_2_S (424.98).

#### *1-Acetyl-5-fluoro-3-((thiophen-2-ylmethylene)hydrazono)indolin-2-one (*6*)*

Yield 84%, yellow solid, m.p.: 175–178 °C. IR: 1710, 1725, and 3100 cm^−1^: ^1^H NMR (700.170 MHz, DMSO-*d*_6_): 2.64 (s, 3H, CH_3_), 7.35 (t, 1H, *J* = 14 Hz, thiaz-H), 7.48 (t, 1H, *J* = 14 Hz, Ph), 7.91–7.97 (m, 1H, thiaz-H), 8.08–8.11 (m, 2H, Ph, thiaz-H), 8.39–8.43 (m, 1H, CH), and 9.05 (s, 1H, Ph). ^13^C NMR (DMSO-*d*_6_) *δ* = 26.2, 111.9, 115.4, 120.1, 129.2 (2×), 134 (2×), 136.1 (2×), 141.3, 151.1, 156.9, 164.6, and 169.7.

MS (ESI, 70 eV): *m/z* 316 (MH)^+^. Anal. Calcd. for C_15_H_10_FN_3_O_2_S (315.05).

#### *2-(2-Oxoindolin-3-ylidene)hydrazinethiocarbohyzrazide (*7*)*

Yield 73%, brown solid, m.p.: 278–280 °C. IR: 1710, 3100, and 3100 cm^−1^: ^1^H NMR (700.170 MHz, DMSO-*d*_6_): 6.91–6.99 (m, 1H, Ph), 7.17–7.21 (m, 2H, Ph), 7.59–7.63 (m, 1H, Ph), 7.61–7.64 (m, 1H, Ph), 11.33 (br s, 2H, NH_2_), 13.01 (br s, 1H, NH), and 14.95 (br s, 1H, NH). ^13^C NMR (DMSO-*d*_6_) *δ* = 112.6, 119.9, 121.1, 138.8, 151.1, 159.8, 163.1, 175.7, and 184.8.

MS (ESI, 70 eV): *m/z* 236 (MH)^+^. Anal. Calcd. for C_9_H_9_N_5_OS (235.05).

#### *3-((4-(3,4,5-Trihydroxy-6-(hydroxymethyl)tetrahydro-2H-pyran-2-yloxy)benzylidene) hydrazono)indolin-2-one (*8*)*

Yield 71%, light yellow solid, m.p.: 146–149 °C. IR: 1715, 3100, and 3500 cm^−1^: ^1^H NMR (700.170 MHz, DMSO-*d*_6_): 3.35–3.41 (m, 3H, 3 × H), 3.77–3.81 (m, 3H, OH, 2 × CH), 3.99 (s, 1H, OH), 4.55–4.61 (m, 1H, CH), 4.74–4.81 (m, 1H, CH), 5.11–5.15 (m, 1H, CH), 5.27–5.30 (m, 2H, CH_2_), 6.99 (s, 1H, Ph), 7.16 (s, 1H, Ph), 7.31–7.35 (m, 2H, Ph), 7.57–7.62 (m, 1H, Ph), 8.11–8.15 (m, 3H, Ph), 8.41 (s, 1H, CH), and 10.91 (br s, 1H, NH). ^13^C NMR (DMSO-*d*_6_) *δ* = 61.3, 67.4, 70.6, 71.9, 75.2, 98.5, 111.2, 117, 117.1, 122.8, 127.4, 129.4, 131.3 (2×), 134 (2×), 145.3, 151.2, 161.2, 162.3, and 165.2.

MS (ESI, 70 eV): *m/z* 428 (MH)^+^. Anal. Calcd. for C_21_H_21_N_3_O_7_ (427.14).

#### *3-(((9H-Fluoren-2-yl)methylene)hydrazono)indolin-2-one (*9*)*

Yield 64%, yellow solid, m.p.: 181–183 °C. IR: 1715, 1725, and 3100 cm^−1^: ^1^H NMR (700.170 MHz, DMSO-*d*_6_): *δ* = 4.08 (s, 2H, CH_2_), 6.93 (d, 1H, Ph, *J* = 7 Hz), 7.09 (s, 1H, Ph), 7.43 (dd, *J* = 7 Hz, 3H, Ph), 7.68 (d, 1H, *J* = 13.8 Hz, Ph), 8.03 (d, 3H, *J* = 14 Hz, Ph), 8.12 (s, 1H, CH), 8.22 (s, 1H, Ph), 8.76 (s, 1H, Ph), and 10.90 (s, 1H, br s, NH). ^13^C NMR (DMSO-*d*_6_) *δ* = 36.9, 111.3, 117, 121.1, 121.4, 122.9, 125.8, 125.9, 127.5, 128.4, 128.8, 129.3, 132.4, 134.1, 140.6, 144.3, 144.6, 145.4, 145.6, 151, 162.3, and 165.1.

MS (ESI, 70 eV): *m/z* 338 (MH)^+^. Anal. Calcd. for C_22_H_15_N_3_O (337.12).

#### *3-(((9H-Fluoren-2-yl)methylene)hydrazono)-1-acetylindolin-2-one (*10*)*

Yield 82%, light yellow solid, m.p.: 162–165 °C. IR: 1712, 1725, and 3150 cm^−1^: ^1^H NMR (700.170 MHz, DMSO-*d*_6_): 2.66 (s, 3H, CH_3_), 4.08 (s, 2H, CH_2_), 7.40 (dd, 3H, *J* = 14 Hz, Ph), 7.60 (dd, 2H, *J* = 14 Hz, Ph), 8.04–8.09 (m, 3H, CH, Ph), 8.08 (d, 2H, *J* = 7 Hz, Ph), 8.25–8.31 (m, 1H, Ph), and 8.84 (s, 1H, Ph). ^13^C NMR (DMSO-*d*_6_) *δ* = 27, 36.9, 116.8, 118.3, 121.2, 121.5, 125.8, 125.9, 126.2, 127.5, 128.6, 128.9, 129.2, 132.2, 133.9, 140.5, 142.9, 144.4, 144.7, 145.9, 149.1, 163.7, 164.1, and 170.7.

MS (ESI, 70 eV): *m/z* 380 (MH)^+^. Anal. Calcd. for C_24_H_17_N_3_O_2_ (379.13).

#### *3-(((2,3-Dihydrobenzo[b][1,4]dioxin-6-yl)methylene)hydrazono)indolin-2-one (*11*)*

Yield 64%, yellow solid, m.p.: 177–180 °C. IR: 1715, 3100 cm^−1^: ^1^H NMR (700.170 MHz, DMSO-*d*_6_): 4.37 (d, 4H, *J* = 14 Hz, 2 × CH_2_), 6.91 (t, 1H, *J* = 14 Hz, Ph), 7.09 (t, 2H, *J* = 7 Hz, Ph), 7.40–7.53 (m, 3H, Ph), 7.99 (d, 1H, *J* = 14 Hz, Ph), 8.58 (d, 1H, *J* = 14 Hz, Ph), and 10.85 (br s, 1H, NH). ^13^C NMR (DMSO-*d*_6_) *δ* = 64.4, 65, 111.2, 117, 117.7, 118.4, 122.8, 123.4, 127.3, 129.2, 134, 144.2, 145.3, 147.7, 151.1, 161.8, and 165.1.

MS (ESI, 70 eV): *m/z* 308 (MH)^+^. Anal. Calcd. for C_17_H_13_N_3_O_3_ (307.10).

#### *5-Chloro-3-(((2,3-dihydrobenzo[b][1,4]dioxin-6-yl)methylene)hydrazono)indolin-2-one (*12*)*

Yield 84%, yellow solid, m.p.: 183–186 °C. IR: 1710 and 3150 cm^−1^: ^1^H NMR (700.170 MHz, DMSO-*d*_6_): 4.36 (q, 4H, 2 × CH_2_), 6.93 (d, 1H, *J* = 7 Hz, Ph), 7.10 (d, 1H, *J* = 14 Hz, Ph), 7.48 (dd, 3H, *J* = 14 Hz, *J* = 6.8 Hz, Ph), 7.98 (s, 1H, Ph), 8.64 (s, 1H, CH), and 11.01 (br s, 1H, NH). ^13^C NMR (DMSO-*d*_6_) *δ* = 64.4, 65.1, 112.8, 117.7, 118.2, 118.5, 122.6, 126.2, 127.1, 128.5, 133.4, 144.1, 144.3, 148.1, 150.6, 163.6, and 164.8.

MS (ESI, 70 eV): *m/z* 343 (MH)^+^. Anal. Calcd. for C_17_H_12_ClN_3_O_3_ (341.06).

#### *1-Acetyl-3-(((1-acetyl-1H-indol-3-yl)methylene)hydrazono)indolin-2-one (*13*)*

Yield 78%, yellow solid, m.p.: 163–166 °C. IR: 1715, 1730, and 3050 cm^−1^: ^1^H NMR (700.170 MHz, DMSO-*d*_6_): 2.67 (s, 3H, CH_3_), 3.35 (s, 3H, CH_3_), 7.37 (d, 1H, *J* = 7 Hz, Ph), 7.53–7.59 (m, 2H, Ph), 7.61 (t, 1H, *J* = 14 Hz, *J* = 14 Hz, Ph), 8.30 (d, 1H, *J* = 7 Hz, Ph), 8.43–8.50 (m, 3H, CH, Ph), 8.74 (s, 1H, Ph), and 8.90 (s, 1H, Ph). ^13^C NMR (DMSO-*d*_6_) *δ* = 24.3, 27.1, 116.8, 116.9, 118.3, 122.1, 125.4, 125.7, 126.7 (2×), 127.8, 133.8 (2×), 136.6, 136.8, 142.7, 148.3, 158.5, 164.1, 170.3, and 170.7.

MS (ESI, 70 eV): *m/z* 373 (MH)^+^. Anal. Calcd. for C_21_H_16_N_4_O_3_ (372.12).

#### *3-(2-Hydroxy-1,2-diphenylethylimino)indolin-2-one (*14*)*

Yield 78%, red solid, m.p.: 159–162 °C. IR: 1710, 3300, and 3400 cm^−1^: ^1^H NMR (700.170 MHz, DMSO-*d*_6_): 6.03 (d, 1H, *J* = 4 Hz, CH), 6.09 (d, 1H, *J* = 4 Hz, CH), 6.94 (d, 1H, *J* = 7 Hz, Ph), 7.26–7.31 (m, 1H, Ph), 7.32–7.37 (m, 2H, Ph), 7.38–7.41 (m, 2H, Ph), 7.42–7.48 (m, 3H, Ph), 7.58 (d, 2H, *J* = 14 Hz, Ph), 7.63–7.70 (m, 2H, Ph), 8.01–8.06 (m, 2H, Ph), and 11.14 (br s, 1H, NH). ^13^C NMR (DMSO-*d*_6_) *δ* = 71.1, 76.1, 114.3, 124.6, 127.2 (2×), 127.7, 128.1 (2×), 128.9, 129.1 (3×), 129.3 (2×), 133.7, 135.1, 137.7, 149.6, 159.6, 183.8, and 199.6.

MS (ESI, 70 eV): *m/z* 343 (MH)^+^. Anal. Calcd. for C_22_H_18_N_2_O_2_ (342.14).

#### *9-Ethyl-1,3-dimethyl-8-(4-(2-oxoindolin-3-ylideneamino)phenoxy)-1H-purine-2,6(3H,9H)-dione (*15*)*

Yield 71%, brown solid, m.p.: 167–169 °C. IR: 1715, 1735, and 3100 cm^−1^: ^1^H NMR (700.170 MHz, DMSO-*d*_6_): 1.41 (t, 3H, *J* = 14 Hz, *J* = 14 Hz, CH_3_), 3.25 (s, 6H, *N*-CH_3_), 4.27 (d, 2H, *J* = 7 Hz, CH_2_), 6.92 (d, 2H, *J* = 14 Hz, Ph), 7.03 (d, 2H, *J* = 14 Hz, *J* = 14 Hz, Ph), 7.43 (d, 2H, *J* = 14 Hz, *J* = 14 Hz, Ph), 7.51–7.56 (m, 1H, Ph), 7.57–7.61 (m, 1H, Ph), and 11.01 (br s, 1H, NH). ^13^C NMR (DMSO-*d*_6_) *δ* = 15.8, 18.1, 39.5 (2×), 111.5 (2×), 111.6 (2×), 116.2, 116.6, 123 (2×), 128.6 (2×), 134.8 (2×), 139.5, 145.2 (2×), 145.6 (2×), 163.8, and 184.8.

MS (ESI, 70 eV): *m/z* 445 (MH)^+^. Anal. Calcd. for C_23_H_20_N_6_O_4_ (444.15).

#### *1-(2-(Methylthio)ethyl)indoline-2,3-dione (*16*)*

Yield 66%, yellow solid, m.p.: 248–250 °C. IR: 1715 and 3100 cm^−1^: ^1^H NMR (500.133 MHz, CDCl_3_): *δ* = 2.19 (s, 3H, SCH_3_), 2.81 (t, 2H, *J* = 14 Hz, CH_2_), 3.95 (t, 2H, *J* = 14 Hz, CH_2_), 6.95 (d, 1H, *J* = 14 Hz, *J* = 7 Hz, Ph), 7.13 (d, 1H, *J* = 7 Hz, Ph), and 7.60 (t, 2H, *J* = 15 Hz, Ph). ^13^C NMR (DMSO-*d*_6_) *δ* = 18.1, 19.7, 62.6, 100.6, 111.3, 120.7, 121.5, 137.1, 142.4, 162.2, and 179.3.

MS (ESI, 70 eV): *m/z* 222 (MH)^+^. Anal. Calcd. for C_11_H_11_NO_2_S (221.05).

#### *5-Fluoro-1-(2-(methylthio)ethyl)indoline-2,3-dione (*17*)*

Yield 55%, red solid, m.p.: 182–184 °C. IR: 1715 and 3040 cm^−1^: ^1^H NMR (700.170 MHz, DMSO-*d*_6_): 2.36 (s, 3H, SCH_3_), 3.25–3.38 (m, 2H, CH_2_), 4.25 (d, 2H, *J* = 7 Hz, CH_2_), 6.95 (d, 1H, *J* = 7 Hz, Ph), 7.25 (d, 1H, *J* = 7 Hz, Ph), and 7.53–7.59 (m, 1H, Ph). ^13^C NMR (DMSO-*d*_6_) *δ* = 15.8, 28.3, 30.1, 111.3, 120.3, 122.9, 132.3, 133.9, 148.5, 151.3, and 165.9.

MS (ESI, 70 eV): *m/z* 240 (MH)^+^. Anal. Calcd. for C_11_H_10_FNO_2_S (239.04).

#### *1-(2-Hydroxyethyl)indoline-2,3-dione (*18*)*

Yield 62%, yellow solid, m.p.: 151–154 °C. IR: 1720 and 3300 cm^−1^: ^1^H NMR (700.170 MHz, DMSO-*d*_6_): 4.36 (t, 2H, *J* = 3 Hz, CH_2_), 4.59 (t, 2H, *J* = 3.1 Hz, CH_2_), 6.86 (d, 1H, *J* = 7 Hz, Ph), 7.09 (t, 1H, *J* = 7 Hz, Ph), 7.32 (d, 1H, *J* = 7 Hz, Ph), 7.37 (t, 1H, *J* = 14 Hz, Ph), and 8.14 (br s, 1H, OH). ^13^C NMR (DMSO-*d*_6_) *δ* = 65.8 (2×), 110.7, 123.3 (2×), 124.3, 125.2, 131.6, 141.7, and 174.4.

MS (ESI, 70 eV): *m/z* 192 (MH)^+^. Anal. Calcd. for C_10_H_9_NO_3_ (191.06).

#### *3,4a,5,9b-Tetrahydro-2H-[1,4]oxathiino[2,3-b]indole (*19*)*

Yield 68%, orange solid, m.p.: 128–130 °C. IR: 1720 and 2900 cm^−1^: ^1^H NMR (700.170 MHz, DMSO-*d*_6_): 3.5 (dd, 2H, 2 × CH), 4.47 (q, 1H, CH), 4.58 (q, 1H, CH), 6.85 (d, 1H, *J* = 7 Hz, Ph), 7.03 (t, 1H, *J* = 14 Hz, Ph), 7.29 (t, 1H, *J* = 14 Hz, Ph), 7.38 (d, 1H, *J* = 7 Hz, Ph), and 10.51 (br s, 1H, NH). ^13^C NMR (DMSO-*d*_6_) *δ* = 34.1, 72.7, 88.5, 110.7, 122.8, 125.8, 127.1, 131.3, 142.1, and 176.9.

MS (ESI, 70 eV): *m/z* 208 (MH)^+^. Anal. Calcd. for C_10_H_9_NO_2_S (207.04).

#### *5-Bromo-4a,5-dihydro-[1,4]oxazino[2,3-b]indole (*20*)*

Yield 66%, light brown solid, m.p.: 115–118 °C. IR: 3100 cm^−1^: ^1^H NMR (700.170 MHz, DMSO-*d*_6_): 7.50 (t, 1H, *J* = 3.5 Hz, CH), 7.76–7.81 (m, 1H, Ph), 7.88–7.91 (m, 2H, CH, Ph), and 7.96 (d, 2H, *J* = 14 Hz, Ph). ^13^C NMR (DMSO-*d*_6_) *δ* = 117.5, 125.2, 127.4, 130.3 (2×), 135.1, 136.2, 145.5, 158.2, and 182.9.

MS (ESI, 70 eV): *m/z* 249 (MH)^+^. Anal. Calcd. for C_10_H_6_BrN_2_O (248.97).

### Anticancer activity study

The anticancer activity of the synthesized compound was tested against 60 different cell lines by the NCI. Briefly, the compound is prepared in DMSO:glycerol, 9:1 at 4 mmol and kept frozen prior to use. Generally, for inoculation, a 96-well microtiter is used for the study of different cell lines. Based on the doubling time for each cell line, the well density varies. Then, the plates are incubated at 37 °C, 5% CO_2_, 95% air, and 100% relative humidity for one day. Later, at 5-log *M* concentration range the tested compound and the control are added to the plates. After two more days, the plates are fixed and stained to identify growth inhibition relative to cells without drug treatment and determination of cell kill as well as net growth inhibition based on time zero control.

### Antibacterial studies

Antimicrobial activity of selected compounds was done by Co-Add Australia against following strains: *Staphylococcus aureus*, *Escherichia coli*, *Klebsiella pneumoniae*, *Pseudomonas aeruginosa*, *Acinetobacter baumannii*, *Candida albicans*, and *Cryptococcus neoformans var. grubii.* Samples were stored frozen at −20 °C, prepared in DMSO and water to a final testing concentration of 32 μg/ml or 20 μM (unless otherwise indicated in the data sheet), in 384-well, non-binding surface plate (NBS) for each bacterial/fungal strain, and in duplicate (*n* = 2), and keeping the final DMSO concentration to a maximum of 1% DMSO. All the sample preparation was done using liquid handling robots.

### Molecular docking calculation

An important method used to determine the molecules with the highest activity against biological materials is docking. Molecular docking calculations are made by Schrödinger’s Maestro Molecular modelling platform (version 12.8)^35–37^. In the calculations made with this method, it is possible to comment on the active sites of the molecules. Calculations are made up of several steps. It first uses the protein preparation module^38^ to prepare the protein, then the LigPrep module^39^ to prepare the molecules. Prepared proteins and molecules are interacted with each other with the Glide ligand docking tool^40^. Finally, the Qik-prop module of the Schrödinger software^41^ was used while performing ADME/T analysis in order to examine the effects and effects of the studied molecules on human metabolism.

## Conclusions

These results encourage the potential implementation of isatin derived analogs as application in cancer treatment by inhibition of cancer cell lines. Some of the molecules (**3**, **4**, and **19**) showed good to excellent antiproliferative activity against renal cancer cell lines and their docking scores are higher as compared to others. Moreover, compound **15** exhibited excellent activity against CNS cancer cell line selectively. The synthesized molecules could further be derivatized and modified for maximum potency and less toxicity. Moreover, the potential efficacy and safety of high doses of synthesized compounds should also be critically assessed by further larger *in vivo*/human studies.

## Supplementary Material

Supplemental Material
