# Supplementary material for: Synthesis biological evaluation and molecular docking of isatin hybrids as anti-cancer and anti-microbial agents
Source: J Enzyme Inhib Med Chem. 2023 Dec 11;39(1):2288548. doi: 10.1080/14756366.2023.2288548 (PMC11721758; doi:10.1080/14756366.2023.2288548)
Supplement: Supplemental Material [file IENZ_A_2288548_SM9200.pdf]

S1. Numerical values of the docking parameters of molecule against enzymes

**1JIJ**	**Docking Score**	**Glide ligand efficiency**	**Glide hbond**	**Glide evdw**	**Glide ecoul**	**Glide emodel**	**Glide energy**	**Glide einternal**	**Glide posenum**
**3**	-4.56	-0.18	-0.48	-31.24	-9.44	-50.66	-40.68	1.01	359
**4**	-6.33	-0.28	-0.55	-43.44	-6.75	-67.69	-50.19	1.95	383
**5**	-4.56	-0.18	-0.48	-31.24	-9.44	-50.66	-40.68	1.01	359
**6**	-5.98	-0.27	-0.14	-44.69	-6.22	-68.90	-50.90	4.51	345
**7**	-6.19	-0.39	-0.16	-33.31	-11.12	-68.64	-44.43	0.19	289
**8**	-6.31	-0.20	-0.16	-39.50	-18.73	-77.37	-58.23	11.70	35
**9**	-6.73	-0.26	0.00	-44.75	-5.77	-71.56	-50.52	1.54	164
**10**	-4.55	-0.16	-0.25	-39.34	-4.00	-55.09	-43.34	1.84	173
**11**	-6.50	-0.28	-0.01	-43.14	-7.32	-69.84	-50.46	1.47	149
**12**	-8.76	-0.28	-0.01	-45.30	-6.41	-72.64	-51.71	1.22	135
**13**	-8.42	-0.34	-0.33	-50.59	-9.62	-88.80	-60.21	2.10	376
**14**	-7.49	-0.29	-0.39	-37.06	-12.36	-71.49	-49.43	4.16	8
**15**	-5.25	-0.16	-0.17	-44.56	-5.70	-68.71	-50.26	1.11	80
**16**	-6.87	-0.46	-0.32	-32.78	-7.90	-57.77	-40.68	0.96	393
**17**	-5.59	-0.35	0.00	-31.48	-8.26	-53.10	-39.73	1.03	14
**18**	-7.19	-0.51	-0.72	-27.41	-11.48	-55.79	-38.88	2.41	365
**19**	-5.40	-0.39	0.00	-27.27	-2.03	-39.74	-29.31	0.00	47
**20**	-6.10	-0.44	0.00	-33.34	-4.51	-55.19	-37.85	0.00	122
**4HKG**	**Docking Score**	**Glide ligand efficiency**	**Glide hbond**	**Glide evdw**	**Glide ecoul**	**Glide emodel**	**Glide energy**	**Glide einternal**	**Glide posenum**
**3**	-4.03	-0.16	0.00	-25.59	-1.57	-33.17	-27.16	0.50	38
**4**	-4.00	-0.17	0.00	-24.83	-1.77	-33.31	-26.60	0.04	324
**5**	-4.03	-0.16	0.00	-25.59	-1.57	-33.17	-27.16	0.50	38
**6**	-4.21	-0.19	0.00	-21.78	-1.25	-28.66	-23.04	0.48	234
**7**	-4.25	-0.27	-0.16	-19.57	-3.18	-27.56	-22.76	2.97	189
**8**	-4.37	-0.14	-0.14	-21.19	-12.51	-38.78	-33.70	9.32	67
**9**	-4.49	-0.17	-0.32	-26.13	-3.65	-37.07	-29.77	2.05	203
**10**	-	-	-	-	-	-	-	-	-

**11**	-4.66	-0.20	-0.32	-23.46	-3.91	-34.51	-27.37	0.50	355
**12**	-4.48	-0.19	-0.32	-23.64	-3.44	-33.99	-27.08	0.17	2
**13**	-3.86	-0.15	-0.21	-20.37	-2.66	-28.89	-23.03	0.26	390
**14**	-4.08	-0.16	0.00	-24.26	-0.92	-29.67	-25.18	2.31	386
**15**	-3.48	-0.11	-0.47	-28.78	-11.78	-46.17	-40.56	5.43	108
**16**	-4.89	-0.30	0.00	-16.96	-2.45	-23.69	-19.40	2.70	309
**17**	-4.63	-0.29	0.00	-17.98	-2.51	-25.43	-20.50	2.26	365
**18**	-4.57	-0.33	0.00	-17.44	-2.30	-24.64	-19.74	1.47	99
**19**	-4.85	-0.35	-0.32	-14.96	-2.66	-22.60	-17.62	0.00	168
**20**	-4.41	-0.32	0.00	-15.24	-3.32	-26.31	-18.56	0.00	53
**1T9U**	**Docking Score**	**Glide ligand efficiency**	**Glide hbond**	**Glide evdw**	**Glide ecoul**	**Glide emodel**	**Glide energy**	**Glide einternal**	**Glide posenum**
**3**	-5.10	-0.20	-0.32	-32.18	-8.97	-53.03	-41.15	2.42	199
**4**	-4.63	-0.20	-0.16	-31.31	-4.94	-46.33	-36.25	0.07	230
**5**	-5.10	-0.20	-0.32	-32.18	-8.97	-53.03	-41.15	2.42	199
**6**	-5.13	-0.23	-0.18	-28.86	-4.82	-44.62	-33.68	2.24	112
**7**	-4.70	-0.29	-0.86	-20.64	-8.62	-37.13	-29.25	2.82	342
**8**	-8.04	-0.26	-0.73	-41.72	-16.68	-69.03	-58.40	37.47	118
**9**	-3.96	-0.15	-0.24	-27.40	-5.31	-40.42	-32.71	0.42	104
**10**	-	-	-	-	-	-	-	-	-
**11**	-5.92	-0.26	-0.63	-27.31	-7.72	-47.25	-35.03	2.65	335
**12**	-8.67	-0.24	-0.57	-29.91	-7.69	-49.94	-37.61	0.93	129
**13**	-4.66	-0.19	-0.65	-25.44	-6.87	-40.46	-32.31	3.24	354
**14**	-5.32	-0.20	-0.48	-32.08	-6.91	-48.08	-38.99	7.51	245
**15**	-4.14	-0.13	-0.67	-30.02	-7.60	-47.88	-37.62	3.49	37
**16**	-5.22	-0.35	-0.41	-23.32	-4.20	-36.32	-27.52	1.10	202
**17**	-4.21	-0.26	-0.21	-20.26	-4.56	-31.01	-24.82	1.48	323
**18**	-6.08	-0.43	-0.91	-17.80	-9.74	-37.60	-27.54	2.41	10
**19**	-4.62	-0.33	-0.13	-21.88	-3.88	-33.57	-25.77	0.00	97
**20**	-4.74	-0.34	-0.16	-19.30	-3.39	-29.77	-22.69	0.00	119
**2UV0**	**Docking Score**	**Glide ligand efficiency**	**Glide hbond**	**Glide evdw**	**Glide ecoul**	**Glide emodel**	**Glide energy**	**Glide einternal**	**Glide posenum**
**3**	-4.95	-0.19	-0.32	-26.90	-6.99	-41.91	-33.89	3.31	333

**4**	-3.58	-0.16	0.00	-28.24	-2.08	-34.68	-30.32	3.87	276
**5**	-4.95	-0.19	-0.32	-26.90	-6.99	-41.91	-33.89	3.31	333
**6**	-3.63	-0.16	-0.32	-22.22	-7.18	-32.52	-29.40	2.29	279
**7**	-3.73	-0.23	-0.03	-17.76	-9.18	-36.17	-26.94	1.30	93
**8**	-5.89	-0.19	-0.62	-29.23	-14.07	-54.69	-43.30	6.97	329
**9**	-5.37	-0.21	-0.25	-28.75	-6.21	-44.63	-34.96	2.58	321
**10**	-5.82	-0.20	-0.32	-29.31	-6.78	-46.33	-36.09	4.54	135
**11**	-4.45	-0.19	-0.32	-22.53	-5.20	-34.59	-27.73	1.05	352
**12**	-4.88	-0.20	-0.48	-23.89	-3.93	-34.96	-27.81	2.02	273
**13**	-4.46	-0.18	-0.39	-25.46	-4.16	-37.20	-29.62	1.72	139
**14**	-4.51	-0.17	-0.31	-27.15	-4.31	-38.27	-31.45	2.06	310
**15**	-4.11	-0.13	-0.69	-29.37	-5.92	-44.91	-35.29	1.76	256
**16**	-3.90	-0.26	-0.15	-16.84	-4.42	-26.86	-21.27	1.46	193
**17**	-3.69	-0.23	-0.08	-18.99	-4.17	-28.97	-23.16	0.56	165
**18**	-4.31	-0.31	-0.40	-17.23	-6.44	-30.02	-23.67	1.77	325
**19**	-4.77	-0.34	-0.32	-15.53	-4.78	-26.65	-20.31	0.00	173
**20**	-3.61	-0.26	0.00	-17.04	-5.63	-29.16	-22.67	0.00	392

S2. ADME properties of molecule

	**3**	**4**	**5**	**6**	**7**	**8**	**9**	**10**	**11**	**Referance Range**
mol_MW	426	384	426	315	235	427	337	379	307	130-725
dipole (D)	6.4	4.0	6.4	5.1	4.7	3.8	3.4	5.3	4.0	1.0-12.5
SASA	656	592	656	575	475	717	650	718	562	300-1000
FOSA	100	18	100	106	20	148	78	162	147	0-750
FISA	98	103	98	105	238	255	102	101	102	7-330
PISA	368	382	368	288	182	313	469	455	313	0-450
WPSA	90	90	90	76	35	0	0	0	0	0-175
volume (A^3)	1145	1013	1145	976	764	1276	1123	1257	969	500-2000
donorHB	0	1	0	0	4	5	1	0	1	0-6
accptHB	5.5	5	5.5	5.5	6	14.2	5	5.5	6.5	2.0-20.0
glob (Sphere =1)	0.8	0.8	0.8	0.8	0.9	0.8	0.8	0.8	0.8	0.75-0.95

QPpolrz (A^3)	41.3	36.1	41.3	33.7	23.0	41.5	40.0	45.3	33.7	13.0-70.0
QPlogPC16	12.8	11.8	12.8	9.9	9.3	15.3	12.9	13.9	10.3	4.0-18.0
QPlogPoct	17.5	16.7	17.5	14.6	17.2	29.3	17.6	18.3	16.2	8.0-35.0
QPlogPw	9.3	10.2	9.3	8.6	14.4	24.5	10.5	9.5	11.1	4.0-45.0
QPlogPo/w	4.2	3.6	4.2	2.9	-0.5	-0.2	4.1	4.6	2.3	-2.0-6.5
QPlogS	-5.7	-5.2	-5.7	-4.1	-1.1	-3.1	-5.5	-6.1	-3.7	-6.5-0.5
CIQPlogS	-6.7	-6.1	-6.7	-4.1	-1.2	-3.3	-5.3	-5.8	-3.9	-6.5-0.5
QPlogHERG	-6.4	-6.2	-6.4	-5.7	-5.5	-6.4	-6.9	-7.2	-5.6	*
QPPCaco (nm/sec)	1163	1047	1163	999	14	38	1070	1085	1070	**
QPlogBB	-0.4	-0.4	-0.4	-0.5	-1.4	-2.7	-0.8	-0.8	-0.6	-3.0-1.2
QPPMDCK (nm/sec)	1806	1612	1806	1291	8	14	532	540	532	**
QPlogKp	-1.7	-1.8	-1.7	-2.2	-6.8	-4.2	-1.3	-1.3	-2.0	Kp in cm/hr
IP (ev)	8.7	8.6	8.7	8.8	8.3	8.7	8.7	8.8	8.7	7.9-10.5
EA (eV)	1.6	1.5	1.6	1.5	1.1	1.0	1.0	1.1	1.0	-0.9-1.7
#metab	2	2	2	2	1	5	1	1	1	1-8
QPlogKhsa	0.3	0.3	0.3	-0.1	-0.6	-0.9	0.5	0.6	-0.1	-1.5-1.5
Human Oral Absorption	3	3	3	3	2	2	3	3	3	-
Percent Human Oral Absorption	100	100	100	100	44	54	100	100	95	***
PSA	84	70	84	83	134	168	71	85	87	7-200
RuleOfFive	0	0	0	0	0	0	0	0	0	Maximum is 4
RuleOfThree	0	0	0	0	1	0	0	1	0	Maximum is 3
Jm	0.0	0.0	0.0	0.2	0.0	0.0	0.1	0.0	0.6	-

	**12**	**13**	**14**	**15**	**16**	**17**	**18**	**19**	**20**	**Referance Range**
mol_MW	342	330	342	432	221	239	191	207	-	**130-725**
dipole (D)	1.7	3.5	4.8	9.0	5.5	4.9	4.9	4.7	-	**1.0-12.5**
SASA	587	605	607	735	461	470	402	406	-	**300-1000**
FOSA	147	100	7	180	155	155	78	111	-	**0-750**
FISA	102	141	107	226	102	102	158	97	-	**7-330**
PISA	266	364	494	329	166	128	166	169	-	**0-450**
WPSA	72	0	0	0	38	84	0	30	-	**0-175**
volume (A3)	1015	1067	1094	1296	756	771	650	663	-	**500-2000**
donorHB	1	1	2	2	0	0	1	1	-	**0-6**
accptHB	6.5	8	5.2	8.5	5.5	5.5	6.7	4.7	-	**2.0-20.0**

glob (Sphere =1)	0.8	0.8	0.8	0.8	0.9	0.9	0.9	0.9	-	**0.75-0.95**
QPpolrz (A3)	35.1	38.1	38.5	46.9	23.8	24.0	19.5	20.1	-	**13.0-70.0**
QPlogPC16	10.9	11.9	13.1	14.7	7.3	6.9	6.8	6.8	-	**4.0-18.0**
QPlogPoct	16.7	18.7	18.9	24.5	11.0	11.2	11.8	10.7	-	**8.0-35.0**
QPlogPw	10.9	13.3	12.0	15.9	7.4	7.2	10.3	7.9	-	**4.0-45.0**
QPlogPo/w	2.8	2.1	3.7	2.6	1.2	1.4	-0.2	1.2	-	**-2.0-6.5**
QPlogS	-4.5	-3.9	-4.4	-6.0	-1.8	-2.0	-1.1	-1.9	-	**-6.5-0.5**
CIQPlogS	-4.6	-3.8	-5.3	-6.0	-1.8	-2.1	-1.2	-2.2	-	**-6.5-0.5**
QPlogHERG	-5.5	-5.9	-6.4	-6.6	-4.4	-4.3	-3.9	-3.9	-	*****
QPPCaco (nm/sec)	1070	461	963	72	1075	1075	312	1202	-	******
QPlogBB	-0.4	-1.0	-0.8	-2.1	-0.4	-0.3	-0.9	-0.3	-	**-3.0-1.2**
QPPMDCK (nm/sec)	1320	214	475	29	864	1551	141	879	-	******
QPlogKp	-2.2	-2.5	-1.2	-4.2	-2.5	-2.7	-3.6	-2.4	-	**Kp in cm/hr**
IP (ev)	8.8	8.5	9.1	8.1	9.0	9.1	9.4	9.1	-	**7.9-10.5**
EA (eV)	1.1	1.2	0.9	1.0	1.1	1.3	1.0	1.0	-	**-0.9-1.7**
#metab	1	1	4	1	0	0	1	1	-	**1-8**
QPlogKhsa	0.0	-0.2	0.3	0.3	-0.8	-0.7	-0.9	-0.5	-	**-1.5-1.5**
Human Oral Absorption	3	3	3	3	3	3	2	3	-	**-**
Percent Human Oral Absorption	100	87	100	75	88	90	70	89	-	*******
PSA	87	97	72	144	60	60	83	56	-	**7-200**
RuleOfFive	0	0	0	0	0	0	0	0	-	**Maximum is 4**
RuleOfThree	0	0	0	1	0	0	0	0	-	**Maximum is 3**
Jm	0.1	0.1	0.9	0.0	11.7	4.0	4.1	11.2	-	**-**

* corcern below -5, **<25 is poor and >500 is great, *** <25% is poor and >80% is high.

S 3. Numerical values of the docking parameters of molecule against anticancer enzymes

1A06	Docking Score	Glide ligand efficiency	Glide hbond	Glide evdw	Glide ecoul	Glide emodel	Glide energy	Glide einternal	Glide posenum
3	-5.32	-0.28	0.00	-30.34	-1.79	-41.80	-32.14	2.10	373
4	-5.27	-0.23	0.00	-37.31	-0.91	-49.92	-38.21	3.10	279
5	-6.08	-0.23	-0.32	-39.29	-5.79	-59.82	-45.07	3.13	384
6	-6.48	-0.24	0.00	-34.76	-2.64	-47.58	-37.40	2.45	311
7	-5.35	-0.33	-0.55	-20.07	-10.47	-46.00	-30.54	1.91	283
8	-6.24	-0.20	-0.31	-28.20	-19.53	-65.26	-47.73	4.87	74
9	-6.41	-0.25	0.00	-39.97	-2.05	-56.89	-42.02	3.37	374
10	-6.41	-0.22	0.00	-43.53	-2.30	-62.66	-45.83	2.36	342
11	-5.87	-0.26	0.00	-35.73	-2.31	-51.06	-38.04	1.66	266
12	-6.07	-0.25	0.00	-36.64	-2.16	-52.45	-38.80	1.80	197
13	-5.23	-0.21	0.00	-37.49	-2.49	-49.67	-39.98	5.41	286
14	-4.91	-0.19	-0.13	-27.16	-6.02	-42.80	-33.19	2.86	117
15	-	-	-	-	-	-	-	-	-
16	-	-	-	-	-	-	-	-	-
17	-	-	-	-	-	-	-	-	-
18	-	-	-	-	-	-	-	-	-
19	-4.86	-0.35	-0.15	-20.78	-4.43	-33.51	-25.22	0.00	30
20	-4.60	-0.33	-0.03	-23.31	-0.13	-31.98	-23.44	0.00	177
**6BW2**	**Docking Score**	**Glide ligand efficiency**	**Glide hbond**	**Glide evdw**	**Glide ecoul**	**Glide emodel**	**Glide energy**	**Glide einternal**	**Glide posenum**
3	-8.0	-0.4	0.0	-30.4	0.1	-45.7	-30.4	1.1	154
4	-8.0	-0.3	0.0	-39.9	0.6	-56.7	-39.3	3.6	340
5	-6.2	-0.2	0.0	-37.3	-1.8	-53.4	-39.2	0.6	280
6	-8.8	-0.4	0.0	-43.5	0.2	-65.4	-43.3	3.6	34
7	-6.8	-0.4	-0.2	-33.7	-0.9	-45.8	-34.6	6.1	305
8	-7.5	-0.2	0.0	-30.9	-15.9	-63.0	-46.8	13.0	25
9	-7.1	-0.3	-0.2	-36.9	-4.4	-55.1	-41.3	6.5	396
10	-7.0	-0.2	-0.1	-39.9	-5.7	-60.6	-45.6	5.2	369
11	-6.4	-0.3	0.0	-35.1	-1.4	-50.2	-36.5	1.5	200
12	-6.2	-0.3	0.0	-35.6	-1.3	-50.2	-37.0	1.6	123

13	-7.3	-0.3	0.0	-38.6	-1.7	-56.7	-40.3	3.2	213
14	-8.1	-0.3	-0.2	-31.1	-1.3	-43.4	-32.4	10.3	117
15	-	-	-	-	-	-	-	-	-
16	-	-	-	-	-	-	-	-	-
17	-	-	-	-	-	-	-	-	-
18	-	-	-	-	-	-	-	-	-
19	-7.6	-0.5	0.0	-24.8	-0.9	-37.4	-25.6	0.0	109
20	-6.3	-0.5	0.0	-24.7	-0.1	-36.8	-24.7	0.0	360
